# RaptScore: a large language model-based algorithm for versatile aptamer evaluation

**DOI:** 10.1093/nar/gkaf1480

**Published:** 2026-01-14

**Authors:** Akira Kimura-Yamazaki, Tatsuo Adachi, Shigetaka Nakamura, Yoshikazu Nakamura, Michiaki Hamada

**Affiliations:** Graduate School of Advanced Science and Engineering, Waseda University, Shinjuku-ku Okubo 3-4-1, 169-0072 Tokyo, Japan; RIBOMIC, Minato-ku Shirokanedai 3-16-13, 108-0071 Tokyo, Japan; RIBOMIC, Minato-ku Shirokanedai 3-16-13, 108-0071 Tokyo, Japan; RIBOMIC, Minato-ku Shirokanedai 3-16-13, 108-0071 Tokyo, Japan; Graduate School of Advanced Science and Engineering, Waseda University, Shinjuku-ku Okubo 3-4-1, 169-0072 Tokyo, Japan; Cellular and Molecular Biotechnology (CMB) Research Institute, National Institute of Advanced Industrial Science and Technology (AIST), Koto-ku Aomi 2-3-26, 135-0064 Tokyo, Japan; Graduate School of Medicine, Nippon Medical School, Bunkyo-ku Sendagi1-1-5, 113-8602 Tokyo, Japan

## Abstract

RNA aptamers are a high-potency tool in the life sciences, offering promising applications in drug discovery and beyond. They are typically obtained through systematic evolution of ligands by exponential enrichment (SELEX), which imposes constraints on sequence length and diversity. Several metrics, such as frequency and enrichment, have been developed to identify high-activity aptamers from SELEX. However, existing evaluation metrics are limited to sequences that appear within SELEX and cannot assess sequences of varying lengths, limiting their utility in optimizing aptamer design. To overcome these limitations, we developed RaptScore, a novel binding activity evaluation metric leveraging large language models. RaptScore enables the assessment of arbitrary sequences, including those absent from SELEX, and accommodates variations in sequence length. RaptScore exhibited a strong correlation with binding activity, allowing the identification of shorter aptamers with enhanced binding properties. By integrating RaptScore with *in silico* maturation, we achieved a 10-nucleotide truncation while maintaining binding efficiency. Furthermore, we demonstrated improved aptamer discovery efficiency by combining RaptScore with RaptGen, a variational autoencoder-based aptamer discovery tool. By enabling efficient sequence evaluation and optimization, RaptScore provides a powerful tool for aptamer research, facilitating the discovery of high-activity candidates while reducing experimental effort.

## Graphical Abstract

**Graphical Abstract:**
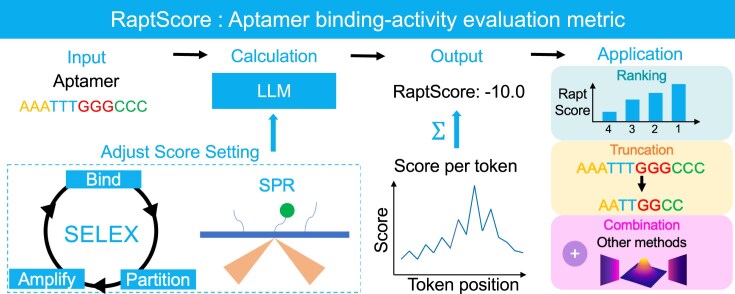

## Introduction

RNA aptamers are single-stranded RNA oligonucleotides that bind to target molecules with high activity and specificity through their unique three-dimensional structures [1]. This property makes RNA aptamers promising tools for a wide range of applications including biosensors [2, 3], diagnostics [4, 5], biomarker [6–8], and therapeutics [9–11]. Aptamers provide multiple benefits compared to conventional protein antibodies. One key advantage is their ability to recognize a diverse array of targets, including metal ions, nucleotides, amino acids, growth factors, various proteins, viral and bacterial pathogens, and whole cells and tissue specimens [12–18]. Aptamers are typically selected experimentally using the SELEX (Systematic Evolution of Ligands by EXponential enrichment) method [19, 20]. SELEX involves iterative rounds of amplification and separation to generate a pool of high-binding-activity aptamers. With advancements in next-generation sequencing (NGS) technologies, high-throughput SELEX (HT-SELEX) [21–23] enables the screening of vast numbers of aptamer candidates. However, identifying aptamers with desirable properties from the enormous sequence space remains a significant challenge. For instance, the sequence space of an RNA aptamer library with 30 random nucleotides is ~ $10^{18}$ ($4^{30}$), while sequencing throughput is ~$10^{6}$. As a result, only a small fraction of the theoretical sequence space can be evaluated, making it extremely difficult to identify aptamers with high activity and specificity for target proteins. In addition, the truncation of aptamer sequences is crucial for commercialization, as it offers two key advantages. The first is a direct reduction in manufacturing and quality assurance costs. The second is an improvement in overall performance and reliability, achieved by mitigating unexpected molecular interactions. Therefore, efficient computational methods to facilitate the discovery of short, high-activity aptamers are essential for aptamer-based drug discovery.

Several computational approaches have been developed to evaluate binding activity from HT-SELEX data. Frequency and enrichment are widely used metrics for ranking aptamer sequences based on occurrence. RaptRanker [24] evaluates aptamers based on sequence and secondary structure similarity. Particle display has also been used for sequence evaluation and truncation, but it requires additional experimental steps beyond SELEX [25]. In addition, several aptamer identification tools [26–28] and simulation-based sequence generation methods [29, 30] are reported. Furthermore, various studies have focused on aptamer sequence generation using methods such as LSTM (long short-term memory networks) [31, 32], VAE (variational autoencoders) [33], and diffusion models [34]. For example, AptaDiff [35] is an aptamer generation model that utilizes diffusion models. RaptGen [36] combines VAE and Bayesian optimization (BO) to generate RNA aptamers. Aptamer discovery methods that combine docking [37] and genetic algorithm (GA) have also been developed [38]. However, docking requires a high computational cost for execution. Despite these advances, existing methods have unresolved limitations. First, many methods cannot evaluate sequences that do not appear in HT-SELEX. For instance, frequency and enrichment rely on SELEX occurrence data, making them unsuitable for novel sequences. Second, these metrics are not suitable for aptamers of varying lengths, as they can only evaluate sequences that are the same length as the target in HT-SELEX. As a result, they are not appropriate for analyzing truncated aptamers.

To address these challenges, we propose RaptScore, an RNA aptamer scoring method that evaluates binding activity using a large language model (LLM) based on transformer architecture [39]. The adaptation of language models to nucleotide sequences has shown significant success [40–43], as demonstrated by studies such as DNABERT [44]. In this work, we leverage large-scale nucleotide language models to assess the naturalness of aptamer sequences, drawing inspiration from research on pseudo-log-likelihood scores (PLLs) [45]. In SELEX, motifs critical for binding activity are selectively amplified and frequently observed in later rounds. When an LLM evaluates such sequences, those containing enriched motifs—indicative of high binding potential—are considered highly natural. Conversely, sequences without these frequently occurring motifs are seen as less natural, implying weaker binding potential. Our approach involves conducting SELEX experiments, measuring the binding activity of a small set of sequences, and using this data to optimize scoring parameters. These refined parameters are then applied to evaluate sequences and guide truncation strategies. Notably, RaptScore can assess sequences of arbitrary lengths, including those not present in SELEX datasets. Through HT-SELEX experiments and surface plasmon resonance (SPR) assays, we demonstrated that RaptScore, when appropriately calibrated, correlates with binding activities measured by SPR. Furthermore, RaptScore facilitated the identification of truncated sequences with improved binding activity and aptamers that retained binding activity even after a 10-nucleotide reduction in length. Additionally, using RaptGen, a VAE-based tool, as an example, we demonstrated that integrating RaptScore with other aptamer discovery tools can further enhance efficiency.

## Materials and methods

### Overview

This section details the methodologies applied in calculating RaptScore. The overall process is as follows. First, SELEX experiments are conducted, generating sequences across five to eight rounds depending on the dataset. Next, three sequences are selected from each SELEX round based on frequency and another three based on enrichment, for a total of six sequences per round, followed by binding activity measurements (e.g. SPR equipment). Subsequently, the optimal RaptScore setting are determined based on their correlation with the measured activity values. RaptScore settings include options such as which SELEX round to use for pretraining and whether to include adapters in the aptamer sequences during pretraining. Using the selected scoring settings, sequence evaluation and truncation are performed. This is made possible by leveraging a transformer-based model, which enables scoring of sequences not present in SELEX and those with varying lengths.

### DNABERT-based transformer model and continual pretraining

We employed DNABERT, a transformer-based deep learning model designed for genomic DNA sequences. DNABERT extends the BERT [46] architecture by incorporating several techniques including *k*-mer tokenization, which enables the model to capture contextual information from nucleotide sequences more effectively. Unlike traditional character-based tokenization, DNABERT represents each DNA sequence as overlapping *k*-mers. For example, a nucleotide sequence “ATGCAT” is tokenized into a series of 3-mers: {ATG, TGC, GCA, CAT}. This approach enhances the contextual understanding of each nucleotide by grouping it with its subsequent bases. Continual pretraining [47] of DNABERT was conducted using SELEX-derived sequence data, employing 3-mer tokenization. The model architecture consists of 12 transformer layers, a hidden dimension of 768, and a total of 86 million parameters. The pretraining process followed the masked language modeling (MLM) approach, where a portion of the input tokens was randomly masked, and the model was trained to predict the original masked tokens based on the surrounding context. Pretraining was conducted using the deep learning framework PyTorch on a server equipped with NVIDIA Tesla V100 GPUs. We generally followed DNABERT’s pretraining strategy and hyperparameters, while reducing the number of training steps to enable faster training.

### RaptScore: a modified PLL score for aptamers

RaptScore is a modified version of the PLL, designed to more effectively evaluate the naturalness of aptamer sequences. RaptScore reflects the “naturalness” of the aptamer sequence within the SELEX data used for pretraining. PLL is computed by systematically masking tokens in a sequence and summing the log probabilities assigned to the masked tokens by a pretrained MLM. This approach provides a bidirectional probability estimate, free from left-to-right biases inherent in autoregressive language models. Given a tokenized sentence or sequence $ W = (w_1, w_2, \dots , w_n)$, the standard PLL is defined as:


\begin{eqnarray*}
\mathrm{PLL}(W) = \sum _{t=1}^{n} \log P_{\mathrm{MLM}}(w_t \mid W_{\backslash t}),
\end{eqnarray*}

where $ W_{\backslash t}$ represents the sequence with the $ t$th token masked and $P_{\mathrm{MLM}}(w_t \mid W_{\backslash t})$ denotes the conditional probability that the MLM assigns to token $w_t$ given the context $W_{\backslash t}$.

In this study, PLL was adapted for aptamers. Each aptamer sequence $ S = (s_1, s_2, \dots , s_L)$ of length $L$ was tokenized into overlapping $k$-mers, resulting in $ W = (w_1, w_2, \dots , w_n)$. Instead of masking a single token at a time, we masked three consecutive tokens as a single unit. Formally, let $M_t = (w_t, w_{t+1}, w_{t+2})$ denote a masked triplet, with masking restricted to $2 \le t \le n-3$. RaptScore is then defined as:


\begin{eqnarray*}
\mathrm{RaptScore}(W) = \sum _{t=2}^{n-3} \log P_{\mathrm{MLM}}(w_{t+1} \mid W_{\backslash M_t}),
\end{eqnarray*}

where $W_{\backslash M_t}$ is the sequence with the triplet $M_t$ masked. As shown in item 5 of the next subsection, an alternative method involves summing the conditional probabilities of the neighboring tokens, $w_t$ and $w_{t+2}$, in addition to the central token, $w_{t+1}$.

This modification was introduced because masking only one token allows the model to infer the masked token from its neighboring tokens, reducing the effectiveness of the score. In addition, to prevent the complete masking of terminal three-nucleotide segments, the masking process starts and ends at the second token from both ends of the sequence. It is important to note that masking a consecutive three-token segment within the sequence corresponds to estimating a single nucleotide, because each nucleotide is covered by three overlapping tokens that include it. In contrast, masking three consecutive tokens from the terminal region results in estimating the entire three-nucleotide sequence at the terminus.

### RaptScore settings and adjustment

**Figure 1.**
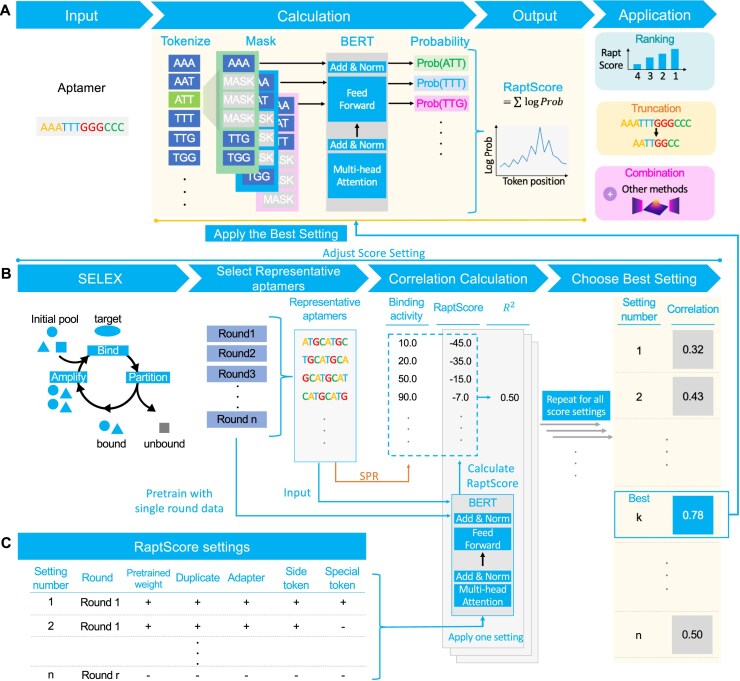
Overview of RaptScore. (**A**) RaptScore is a binding activity metric for RNA aptamers leveraging a language model. Unlike conventional methods, it is applicable to aptamers not included in SELEX and those with varying lengths. RaptScore takes an aptamer sequence as input, tokenizes it, and masks consecutive three-token segments. The model predicts the probability of the masked tokens, and the log likelihoods are summed to compute RaptScore. The optimal scoring settings are selected from multiple candidates. RaptScore can be applied to sequence ranking, truncation, and integration with other aptamer generation methods. (**B**) Evaluation of RaptScore settings. To determine the optimal RaptScore configuration, multiple scoring settings were evaluated using representative aptamers selected from each SELEX round based on frequency and enrichment. The binding activities of these sequences were measured using SPR, and RaptScore values were calculated under each candidate setting. For each setting, the correlation coefficient between RaptScore and binding activity was computed, and the setting with the highest correlation was selected as optimal. (**C**) Setting parameters of RaptScore. The scoring settings include the following parameters: (i) the SELEX round used for pretraining, (ii) whether to use pretrained DNABERT model weight, (iii) how to handle duplicate sequences (keeping them as-is, applying log transformation, or removing duplicates), (iv) whether to include aptamer adapters, (v) whether to add score of tokens other than the central token in masked three-token segments, and (vi) whether to exclude special tokens.

To optimize the RaptScore configuration, various scoring settings were evaluated, and the one yielding the highest correlation coefficient with activity measurements from SPR assays was selected for each dataset. This was used for the following evaluation and analysis (Fig. 1A and B). Correlation was assessed using representative aptamers chosen based on frequency and enrichment. As illustrated in Fig. 1C, the scoring settings were determined by adjusting the following parameters:

1. The SELEX round used for continual pretraining.
2. The initialization of DNABERT model weights, either by further pretraining a pretrained DNABERT model on SELEX data or by pretraining a nonpretrained model from scratch using SELEX data.
3. The treatment of duplicate sequences, which included three approaches: (i) retaining duplicates as-is, (ii) applying a logarithmic transformation to the duplication count $ n$ as $ [\log n]+1$, where $ [\cdot ]$ denotes rounding up to the nearest integer, or (iii) removing duplicate sequences entirely.
4. The inclusion or exclusion of aptamer adapter sequences during training.
5. The calculation of the log-likelihood, either all three masked tokens were summed up, or only the central token was used.
6. The handling of special tokens such as (CLS), (SEP), and (PAD), which could either be included or excluded from the log-likelihood calculation. In cases where the 5 special tokens are excluded, the predicted probabilities are normalized over the remaining 64 tokens to sum to one, and the log-likelihood is then calculated accordingly.

### Truncation based on RaptScore

This section describes the method of truncating sequences based on RaptScore. Parent sequences were selected from those identified by frequency and enrichment as having high experimental binding activity. From each parent sequence, all possible variants with 1–3 nucleotide truncations (~10 000 variants) were generated, and RaptScore was calculated for each. For each length, the top 3–5 scoring sequences were selected, and their binding activities were experimentally measured using SPR. In some datasets, 3–5 sequences with the lowest scores were also selected and measured. For Dataset A, this process was applied to two 35mer sequences. The most active 32-mer sequence was further truncated, ultimately resulting in a 29-mer sequence. For dataset B, the process was similarly applied to two 35-mer sequences. Additionally, combinations of truncation positions (two or three locations) that maintained binding activity after a 1-nucleotide truncation were also evaluated. For Dataset C, the top 5 and bottom 5 sequences with the highest and lowest RaptScore, respectively, were selected from the 1–2 nucleotide truncated variants, and their binding activities were measured, increasing the number to five to assess whether the trend persisted across a wider range of sequences.

### Optimized truncation via RaptScore-guided genetic algorithm

GA were employed for sequence truncation, with the initial sequence pool selected based on RaptScore. Specifically, we extracted the top 100 sequences from Round 4 of Dataset C according to their RaptScore, using these as the initial population for optimization via NSGA-III [48], where RaptScore and aptamer length were set as the two optimization objectives. The initial sequences were 30 nucleotides in length, and the GA was executed for 50 rounds, with crossover and mutation rates set at 0.8 and 0.1, respectively. During mutation, in addition to the standard nucleotides (A, T, G, and C), a special character “X” was introduced to represent truncation sites. From the sequences obtained after 50 iterations, we selected the top-scoring aptamers of lengths 20, 21, and 22 based on RaptScore and further analyzed their secondary structures using RNAfold [49]. To ensure motif conservation, we compared the predicted secondary structures of these candidates with those of the top-scoring sequences from Round 4, and since the length-21 aptamer exhibited structural discrepancies at the predicted motif region, it was excluded. The motif was identified using MEME [50] from the top 20 sequences in Round 4 with the highest RaptScore. Based on this motif analysis and structural screening, two optimized aptamers of lengths 20 and 22 were selected for further experimental validation, and their binding activity was assessed using SPR.

### Selection of potent aptamer via RaptScore and RaptGen integration

RaptGen is a VAE-based aptamer generation model. Representative sequences are selected using Gaussian mixture model (GMM) applied to the latent representations obtained via VAE. Based on the binding activity measurements of the representative sequences, BO is employed to identify sequences with high binding activities. Additionally, truncated sequences can be generated using decoders such as the L20 model and the L25 model, which are specifically designed for producing shortened aptamers. In this study, the combination of RaptGen and RaptScore was evaluated through the following steps. First, the RaptScore settings that showed the best correlation with the binding activity of sequences generated by GMM were selected. These optimized settings were then used to evaluate the correlation coefficients between RaptScore and sequences generated by BO and truncated decoders. Furthermore, the proportion of top 5 binding activity sequences covered by the top 5 sequences ranked by RaptScore was assessed. Two SELEX Datasets, D and E, were used in RaptGen. According to the RaptGen study, 10 sequences were generated and evaluated for each of GMM, BO, and truncated decoder models. The binding activity data reported in the RaptGen study was used for the analysis.

### SELEX and surface plasmon resonance assay

SELEX was performed according to a previously reported method [24, 51] with some modifications. In brief, the target proteins were immobilized on beads. After washing, bound RNA was subsequently retrieved and amplified through reverse-transcription-PCR (polymerase chain reaction). Additional experimental details for the SELEX protocol and the sequencing protocol can be found in Supplementary Text, Supplementary Tables S1–S3.

The SPR assay was performed using a Biacore T200 (Cytiva) [52]. The target proteins of Datasets A and C were human recombinant Fibroblast Growth Factor-9 (R&D systems catalogue no. 273-F9) and Dataset B was mouse recombinant ST2/IL-33R (R&D systems catalogue no. 1004-MR), respectively. Aptamers for Dataset A and C were prepared with primer regions and 16 mer poly(A) tails as follows: $5^{\prime }$-GGGAAGCTCCGTCGAGCT-(variable sequence)-TACGCCTGCGTAGCTCC-poly(A)-$3^{\prime }$ for Dataset A and $5^{\prime }$-GGGA-(variable sequence)-CTCGA-poly(A)-$3^{\prime }$ for dataset for C. Aptamers for Dataset B were prepared with primer region as follows: $5^{\prime }$-GGGAGAACTTCGACT-(variable sequence)-CGTGCAGAGATCCTC-$3^{\prime }$. Aptamers were prepared by *in vitro* transcription using a mutant T7 RNA polymerase an NTPs. The running buffers consisted of 295 mM NaCl, 5.4 mM KCl, 0.8 mM MgCl_2_, 18 mM CaClCl_2_, 0.05% tween 20, and 20 mM Tris–HCl (pH 7.6).

In the case of Dataset A and C, a $5^{\prime }$-Biotinylated dT16 oligomer was immobilized to both active and reference flow cells of the streptavidine sensor chip (BR100531, Cytiva). The poly(A)-tailed RNA was captured in the active flow cell by complementary hybridization at a concentration of 200 nM and flow rate of 20 μl min^−1^, with an association time of 30 s, The proteins were injected into the flow cells of the sensor chip at a concentration of 100 nM and a flow rate of 30 μl min^−1^, with an association time of 60 s. To generate the sensor chip, bound aptamers were completely removed by injecting 6M urea aq.

In the case of Dataset B, a protein A was immobilized to both active and reference flow cell of the CM5 sensor chip (BR100530, Cytiva). The target protein (Fc chimera) was captured in active flow cell by protein A to Fc interaction at a concentration of 50 nM and flow rate of 20 μl min^−1^, with an association time of 30 s. The aptamers were injected into the flow cells of the sensot chip at a concentration of 200 nM and a flow rate 30 μl min^−1^, with an association time of 60 s. To regenerate the sensor chip, bound target proteins were completely removed by injecting 6M GuHCl aq.

Data was obtained by subtracting the reference flow cell data from the active flow cell data. The ratio of the protein-binding level to aptamer-capturing level was used as binding activity. Relative activity was defined as a normalized value, with the control in each experiment set to 100. To validate specificity, a control experiment was conducted using a random sequence, which exhibited negligible binding activity.

## Results

### RaptScore: a computational approach to scoring aptamers

We employed DNABERT, a transformer-based deep learning model designed for genomic DNA sequences for score calculation. Unlike traditional character-based tokenization, DNABERT represents each DNA sequence as overlapping *k*-mers. In our analysis, we used 3-mer tokenization, as larger k values reduce the number of regions where scoring is possible within a sequence, making it unsuitable for shorter sequences. The pretraining process followed the MLM approach using SELEX-derived sequence data based on the strategy used in DNABERT. See the “Materials and methods” section for details on procedures.

**Figure 2.**
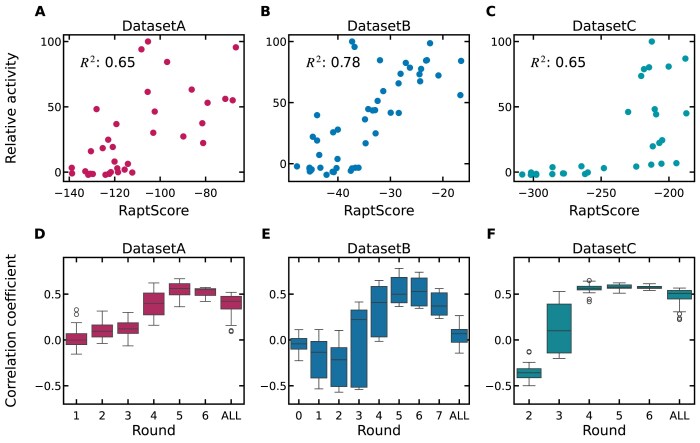
Analysis of correlation between RaptScore and binding activity. Scatter plots showing the correlation between RaptScore and experimentally measured binding activity for Datasets A (**A**), B (**B**), and C (**C**) under the scoring setting that exhibited the highest correlation in each case. For each SELEX round, a total of six representative sequences were selected—three based on frequency and three based on enrichment. Their binding activities were measured using SPR. Relative activity compared to the highest binding sequence selected by frequency or enrichment was shown. Pearson correlation coefficients were calculated to quantify the relationship between RaptScore and binding activity. The scale of RaptScore varies across different settings and datasets, affecting the absolute values of the scores (Supplementary Tables S4–S6). Box plots showing the distribution of correlation coefficients between RaptScore and binding activity across multiple scoring settings for Datasets A (**D**), B (**E**), and C (**F**). Each scoring setting corresponds to a different combination of parameters described in Fig. 1 C, including choices such as the SELEX round used for pretraining, whether to use DNABERT pretrained model weights, and different strategies for handling sequence duplicates. A pooled model trained on the combined data from all SELEX rounds is shown as “ALL” on the *x*-axis. The box indicates the interquartile range, with the line inside representing the median. Whiskers extend to the most extreme data points not considered outliers, and dots represent outliers beyond 1.5× IQR.

We defined a score based on the PLL. PLL reflects the “naturalness” of the aptamer sequence within the SELEX data used for pretraining. Since masking only one or two token allows the model to infer the masked token from its neighboring tokens, we masked three consecutive tokens as a single unit instead of masking a single token at a time. In addition, the masking process starts and ends at the second token from both ends of the sequence. This is because masking three consecutive tokens not at the ends results in only one nucleotide being masked, whereas masking the three tokens at the end leads to the masking of the final three nucleotides. Hereafter, we called this score RaptScore. To optimize the RaptScore configuration, various scoring settings were evaluated, and the one yielding the highest correlation coefficient with activity measurements from SPR assays was selected for each dataset (Fig. 2). This was used for the following evaluation and analysis. Correlation was assessed using representative aptamers chosen based on frequency and enrichment.

For the following evaluations, we used SELEX Datasets A and B, targeting independent proteins, to evaluate the robustness of RaptScore. Dataset C targets the same protein as Dataset A but employs a library with a shorter random region; it was included to examine whether RaptScore can facilitate more effective truncation. Finally, Datasets D and E were directly taken from the previously published RaptGen study and were not newly generated in this work.

### Correlation with binding activity

To evaluate whether RaptScore functions as an indicator of binding activity, we assessed the correlation between RaptScore and experimentally measured binding activity. Sequences were selected from each SELEX round based on frequency and enrichment criteria. Binding activity was measured using SPR. The distribution and correlation coefficients are shown in Fig. 2A–C. The best correlation coefficients between binding activity and RaptScore across all rounds and scoring definitions were 0.65, 0.78, and 0.65 for Dataset A, B, and C, respectively (Supplementary Tables S4–S6).

Further insights into correlation patterns are provided in Fig. 2, which illustrates the distribution of correlation coefficients across different scoring settings for each round. Notably, scores derived from the final SELEX round do not always exhibit the strongest correlation with binding activity. Additionally, we observed consistent variability in correlation coefficients across different scoring definitions within the same round, regardless of the dataset. To examine whether further improvements in performance could be achieved, we also evaluated a pooled model trained on data from all SELEX rounds; however, its correlation coefficients did not surpass those of the best-performing round-specific models (Fig. 2D–F). This suggests that identifying an optimal scoring setting requires a systematic evaluation of both SELEX rounds and scoring definitions to maximize correlation with binding activity.

In summary, RaptScore correlates with binding activity, but the strength of this correlation depends on the SELEX round and scoring settings. Thus, a systematic comparison, as performed above, is necessary to determine the optimal settings. Next, we assessed RaptScore’s ability to select high-activity sequences.

### Comparison of RaptScore with existing aptamer ranking methods

One of the key applications of aptamer binding activity indicators is the selection of sequences with high binding activity. To evaluate whether RaptScore is useful for selecting sequences with high binding activity, we compared the binding activities of sequences selected using three metrics: frequency, enrichment, and RaptScore. Frequency and enrichment are widely used metrics for selecting high-binding aptamers from sequences obtained through SELEX. For SELEX Datasets A, B, and C, the top three sequences were selected from each round using each metric, and their binding activities were measured using SPR.

As shown in Table 1 for Dataset A, sequences selected using RaptScore exhibited higher binding activity than those selected with frequency in Rounds 1–3. Across all rounds, the maximum binding activity of RaptScore-selected sequences was comparable to frequency and higher than enrichment. Table 1 for Dataset B shows that in Dataset B, frequency and enrichment were generally superior across all rounds. Across all rounds, the maximum binding activity of sequences selected with RaptScore was ~90% of the maximum binding activity achieved by sequences selected using frequency and enrichment. According to Table 1 for Dataset C, the maximum binding activity of RaptScore-selected sequences was higher than that of frequency and enrichment across all rounds (Supplementary Tables S7–S9).

**Table 1.** Comparison of the effectiveness of frequency, enrichment, and RaptScore in selecting high-activity aptamers

A									
		Round 1	Round 2	Round 3	Round 4	Round 5	Round 6		All
**Frequency**									
Max		3.32	0.00	27.32	**100.00**	61.45	**94.02**		100.00
Average		0.39	−1.12	12.60	**48.76**	48.86	57.46		-
**Enrichment**									
Max		–^a^	–^b^	–^b^	24.82 ^c^	**95.57**	48.24		95.57
Average		–^a^	–^b^	–^b^	24.82 ^c^	**77.67**	36.87		-
**RaptScore**									
Max		**7.78**	**99.60**	**75.89**	40.41	83.38	92.72 ^d^		99.60
Average		**3.35**	**42.11**	**52.98**	30.29	63.11	**86.81** ^d^		-
**B**									
	**Round 0**	**Round 1**	**Round 2**	**Round 3**	**Round 4**	**Round 5**	**Round 6**	**Round 7**	**All**
**Frequency**									
Max	$-$ 2.20	$-$ 3.31	**22.03**	**73.58**	84.42	**98.59**	78.58	43.83	98.59
Average	$-$ 4.19	$-$ 6.12	**16.05**	37.91	74.13	75.15	65.50	37.41	–
**Enrichment**									
Max	–^a^	−3.30	−3.31	56.11	**84.73**	82.50	**95.59**	**100.00**	100.00
Average	–^a^	−3.30	−4.32	**41.24**	**82.15**	46.91	**68.65**	**64.37**	–
**RaptScore**									
Max	$-$ 1.21	$-$ 4.82	$-$ 4.45	26.33	54.90	87.42	59.41	59.41	87.42
Average	$-$ 2.00	$-$ 5.35	$-$ 5.78	6.47	21.70	**79.07**	17.07	18.15	–
**C**									
			**Round 2**	**Round 3**	**Round 4**	**Round 5**	**Round 6**		**All**
**Frequency**									
Max			**6.85**	−0.99	**100.00**	78.84	**86.93**		100.00
Average			**0.90**	−1.29	**47.83**	43.54	**43.01**		–
**Enrichment**									
Max			−^a^	**80.83**	3.74	80.25	48.17		80.83
Average			−^a^	**51.40**	1.77	43.65	25.69		–
**RaptScore**									
Max			0.89	34.32	57.52	**101.17**	20.70		101.17
Average			0.20	22.46	40.97	**70.96**	10.73		–

Binding activities of aptamers selected using three different ranking methods—frequency, enrichment, and RaptScore—are analyzed across multiple SELEX rounds for each Dataset (A, B, and C). For each round, a total of nine sequences were selected—three based on frequency and three based on enrichment—along with three sequences ranked highest by RaptScore. If fewer than three sequences can be defined based on enrichment, additional sequences are selected based on frequency so that the combined total of sequences selected based on enrichment and frequency remains six. Their binding activities were experimentally measured using SPR to assess how effectively each method identifies high-activity aptamers. Binding activity comparisons for Datasets A (**A**), B (**B**), and C (**C**). The maximum and average binding activities of the selected sequences are reported. Relative activity compared to the highest binding sequence selected by frequency or enrichment was show. Bold formatting highlights the method that identified the highest-activity aptamer in each round (Supplementary Tables S7–S9).

a Enrichment cannot be calculated in the first SELEX round due to the lack of prior rounds for comparison.

b In Dataset A, enrichment could not be calculated for Rounds 2 and 3 because no sequences appeared in consecutive rounds.

c Only one sequence was available for this result.

d RaptScore was calculated based on model pretrained on Round 5 data.

These results suggest that RaptScore demonstrates comparable performance to existing metrics such as frequency and enrichment in identifying high-binding sequences while offering the advantage of adaptability to sequences of any length. Moreover, the results indicate the potential of RaptScore to select high-binding sequences from early SELEX rounds, depending on the dataset. Additionally, unlike enrichment, which can only evaluate sequences that appear in consecutive rounds, RaptScore is not subject to such constraints. Next, we examined whether RaptScore could facilitate sequence truncation.

### Validation of sequence truncation using RaptScore

One potential application of aptamer binding activity indicators is the truncation of aptamer sequences. We evaluated whether RaptScore can facilitate the truncation of aptamer sequences. Notably, existing metrics such as frequency and enrichment, which are commonly used for assessing aptamer binding activity, are only applicable to sequences of the same length as the SELEX target sequences and therefore cannot be utilized for truncation. For the evaluation of binding activity, we selected the top 3–5 sequences with the highest RaptScore and the bottom 3–5 sequences with the lowest RaptScore from each dataset. This strategy was adopted to confirm that sequences with higher scores tend to exhibit stronger binding activity, whereas those with lower scores tend to exhibit weaker binding activity.

**Figure 3.**
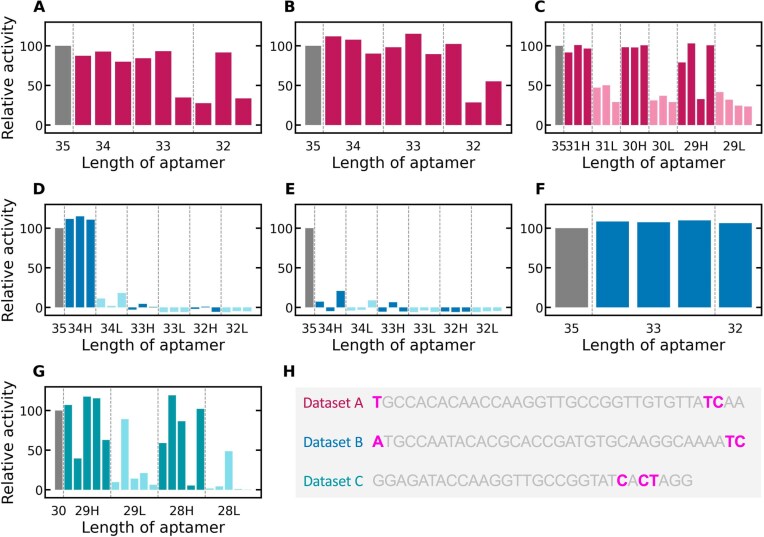
Binding activity and truncation positions of aptamers shortened based on RaptScore. Sequences truncated by 1–3 nucleotides from the original aptamer were selected based on RaptScore, and their binding activities were experimentally measured to assess whether truncation could preserve or improve activity. Relative activity compared to the full-length aptamer was show. Truncation results for Dataset A. (**A, B**) Two different 35-base sequences truncated to 32–34 bases. (**C**) A 32-base sequence truncated to 29–31 bases. The dark-colored bars represent the activity values of sequences with high (H) RaptScore at each length, while the light-colored bars indicate the activity values of sequences with low (L) RaptScore at each length. (D–F) Truncation results for Dataset B. (**D, E**) Two different 35-base sequences truncated to 32–34 bases. (**F**) Alternative truncation strategy applied to a single 35-base sequence, reducing it to 32–34 bases. (**G**) Truncation results for Dataset C. Sequences truncated from 30 to 28–29 bases, derived from a primer-less SELEX dataset. (**H**) Analysis of truncation positions for Datasets A, B, and C (Supplementary Tables S10–S16).

First, for Datasets A and B, RaptScore was calculated for all possible truncated sequences derived from the 35-nt sequences, with 1–3 bases removed. For each length (32–34-nt), the top three sequences based on RaptScore were selected, and their binding activities were measured. Figure 3A and B shows the results of truncation of two 35-nt aptamers from Dataset A. For all lengths (32–34-nt), sequences with higher or comparable binding activity to that of the original 35-nt sequence were obtained (Supplementary Tables S10 and S11).

Next, we explored further truncation of the 32-nt sequence with the highest binding activity. Variants were generated by removing 1–3 nucleotides, resulting in sequences of 29–31 nt in length. We then calculated the RaptScore for each truncated sequence and selected 3–4 sequences with the highest and lowest scores at each length for binding activity measurements. Figure 3C shows the results for these lengths. For all lengths (29–31-nt), sequences with binding activities higher or comparable to the original 35-nt sequence were identified. Moreover, when comparing sequences with high RaptScores to those with low RaptScores, sequences with high RaptScores consistently exhibited superior binding activity across all lengths (29–31-nt) (Supplementary Table S12).

Figure 3D and E illustrates the results of truncation of two aptamers (aptamer B-1 and aptamer B-2) from Dataset B across 32–34-nt lengths. Figure 3D shows that for aptamer B-1, sequences with higher binding activity than the original 35-nt sequence were obtained when truncated by 1 base, but not when truncated by 2 or 3 bases. Figure 3E shows that for aptamer B-2, no high-binding sequences were obtained after truncation by 1, 2, or 3 bases (Supplementary Tables S13 and S14).

Based on these results, we applied a different truncation strategy to aptamer B-1 than the one used for Dataset A. Specifically, 2- or 3-base truncations were performed by combining the truncation sites suggested during the 1-base truncation analysis. Figure 3F presents the outcome of this approach, showing successful truncation in which removing 2 or 3 bases led to improved binding activity compared to the original sequence (Supplementary Table S15). These findings suggest that although truncation strategies may require dataset-specific adjustments, RaptScore can be effectively used to guide aptamer truncation.

To explore further possibilities for truncation, we applied RaptScore to primer-less SELEX data. Dataset C is a SELEX dataset targeting the same molecule as Dataset A but with shorter primer and random regions. Figure 3G presents the results of truncating 30-nt sequences to 28- and 29-nt lengths. For both 28- and 29-nt lengths, sequences with binding activities surpassing the original 30-nt sequences were obtained (Supplementary Table S16). Furthermore, sequences with higher RaptScores tended to exhibit better binding activity compared to those with lower RaptScores. These results suggest that RaptScore can be utilized for truncating sequences while maintaining or improving binding activity, even for primer-less SELEX datasets.

Next, we analyzed which nucleotide positions were truncated in cases where binding activity was maintained after shortening. Figure 3H illustrates the truncation positions for Datasets A, B, and C. Among the truncation candidates identified in panels B, D, and G, only those positions where a single-nucleotide truncation was proposed and the resulting sequence retained >90% of the original binding activity are shown. While truncations were frequently observed at terminal regions, some occurred at internal positions that are difficult to identify through manual inspection. This suggests that RaptScore facilitates the discovery of promising, nonobvious truncation sites and can support more effective sequence optimization. To further assess the utility of RaptScore in sequence truncation, we integrated it with a GA.

### Sequence truncation using the combination of RaptScore and GA

Based on the validations presented so far, RaptScore suggests the potential to estimate aptamer potency independent of laboratory experiments. This notion prompted us to conduct an *in silico* molecular evolution using GA. In the sequential truncation approach described in the previous section, attempting a significant truncation in a single step results in an enormous number of candidate sequences, posing a computational challenge.

**Figure 4.**
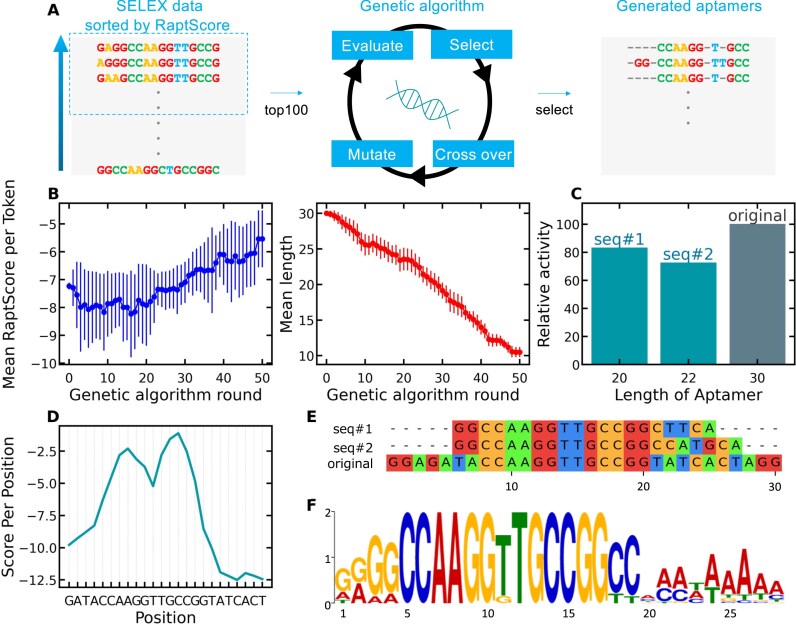
RaptScore-guided GA for aptamer truncation. A GA in combination with RaptScore was used to identify highly truncated aptamer sequences while maintaining binding activity. (**A**) Overview of the GA-based truncation approach. The top 100 sequences ranked by RaptScore from Round 4 of Dataset C were selected as the initial population for GA. Through iterative optimization, truncated sequences were explored. In the figure, “-” indicates the positions where truncation occurred. (**B**) Average RaptScore per token and aptamer length over 50 rounds of the GA. Line plots represent mean values, with error bars indicating the standard deviation at each round. (**C**) Relative activity of GA-generated truncated sequences, experimentally evaluated using SPR. Sequences truncated by 10 bases retained 80% of the binding activity of the untruncated sequences (Supplementary Tables S17). Relative activity compared to the full-length aptamer was show. (**D**) Token-wise RaptScore distribution of the 30-mer sequence with the highest RaptScore from Round 4 of Dataset C. The *x*-axis represents the central nucleotide of each token. The four nucleotides at both termini were excluded due to constraints related to tokenization and score computation. (**E**) Alignment between the original 30-mer sequence and GA-generated truncated sequences. (**F**) Motif analysis results for the top 20 sequences ranked by RaptScore from Round 4 of Dataset C, obtained using MEME.

To address this, we utilized a GA to efficiently explore highly truncated candidates while keeping computational costs manageable. As shown in Fig. 4A, the top 100 sequences with the highest RaptScore from Round 4 of Dataset C were used as the initial population for the GA. Since our goal was to obtain shorter aptamers through truncation, we reasoned that starting from shorter sequences would facilitate generating even shorter candidates. Thus, Dataset C, consisting of aptamers with a length of 30 nucleotides, was selected rather than Datasets A and B, which contain sequences of 35 nucleotides. Figure 4B shows the trends in the average RaptScore per aptamer token and the average aptamer length across 50 rounds of GA. Since the original aptamers were 30 nucleotides in length, we aimed to shorten them by approximately one-third, targeting a reduction of 10 nucleotides. As shown in Fig. 4C, a sequence truncated by 10 bases that retained 80% of the binding activity of the untruncated 30-mer sequences was obtained (Supplementary Table S17). Figure 4D presents the token-wise RaptScore distribution of the 30-mer sequence with the highest RaptScore from Round 4 of Dataset C. The *x*-axis represents the central nucleotide of each token. The four nucleotides at both termini were excluded due to constraints related to tokenization and score computation. Figure 4E shows the alignment between the sequences obtained through GA and the best-ranked sequence from Round 4 of Dataset C based on RaptScore. While some regions were conserved, insertions, mutations, and deletions were observed in nonterminal regions, suggesting that GA was able to explore sequences that would be difficult to identify manually. Figure 4F presents the motif analysis results for the top 20 sequences ranked by RaptScore from Round 4 of Dataset C, obtained using MEME. Compared to Fig. 4D, the RaptScore of the region corresponding to the putative motif CCAAGGTTGCCGG is higher than at other positions. By definition, RaptScore assigns higher scores to subsequences that appear frequently in SELEX, and the results are consistent with this expectation. This finding suggests that position-wise analysis of RaptScore values may aid in motif inference. A comparison between Fig. 4E and F reveals that the putative motif CCAAGGTTGCCGG is preserved before and after truncation by GA. This suggests that the combination of RaptScore and GA enables sequence truncation while preserving important motifs. These results indicate that, depending on the dataset, combining RaptScore with GA enables substantial sequence truncation. Furthermore, to evaluate the applicability of RaptScore when integrated with other aptamer discovery tools, we conducted an analysis combining RaptScore with RaptGen.

### Integration of RaptScore with computational aptamer generation method

**Figure 5.**
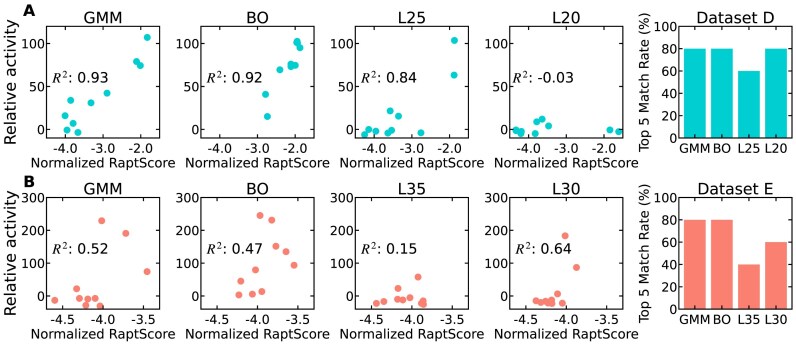
Evaluation of the integration of RaptScore with RaptGen for efficient identification of high-activity aptamers. The analysis was conducted in two stages. First, RaptScore settings were optimized using a small subset of sequences to maximize the correlation with binding activity (Column 1). Second, the optimized settings were applied to assess sequence ranking performance on additional sequences (Columns 2–4). Columns 1–4 show scatter plots comparing RaptScore and experimentally measured binding activity for individual sequences, while Column 5 summarizes the accuracy of RaptScore in identifying top-binding sequences. Note that RaptScore scale is normalized by sequence length to facilitate comparison across aptamers of different lengths. The relative activity data are taken from reported values in the RaptGen study. (**A**) Results for Dataset D. Column 1: 10 sequences selected by GMM, using the RaptScore setting that showed the highest correlation with binding activity ($R^2$ = 0.93). This setting was applied consistently in Columns 2–4. Column 2: Sequences generated by BO ($R^2$ = 0.92). Column 3: Truncated sequences generated by the L25 model (length: 24–30 bases, $R^2$ = 0.84). Column 4: Truncated sequences generated by the L20 model (length: 24–30 bases, $R^2$ = −0.03). Column 5: Proportion of top-binding sequences correctly identified by RaptScore. This represents the fraction of sequences ranked within the top five by RaptScore among those experimentally confirmed to be top five in binding activity (Supplementary Table S18). (**B**) Results for Dataset E. Column 1: 10 sequences selected by GMM ($R^2$ = 0.52), with the same optimized RaptScore setting applied in Columns 2–4. Column 2: BO-generated sequences ($R^2$ = 0.47). Column 3: Sequences generated by the L35 model (length: 38–44 bases, $R^2$ = 0.15). Column 4: Sequences generated by the L30 model (length: 28–40 bases, $R^2$ = 0.64). Column 5: Proportion of top-binding sequences correctly identified by RaptScore (Supplementary Table S19).

To assess whether RaptScore, when combined with aptamer generation tools, can efficiently identify high-binding sequences, we integrated it with RaptGen, a VAE-based aptamer generation method. Generative models such as RaptGen can generate sequences but cannot evaluate the binding activity of arbitrary sequences, posing a significant challenge. By combining generative tools with RaptScore, which can estimate the binding activity of any given sequence, we expect to narrow down candidate sequences for experimental validation while efficiently identifying high-activity aptamers. The evaluation was conducted in two stages. First, using a small subset of sequence data, we optimized RaptScore settings to maximize correlation with binding activity. Then, applying these optimized settings, we tested whether the approach could improve efficiency for identifying high-binding sequences. To evaluate its performance, we analyzed the correlation between RaptScore and experimentally measured binding activity. As shown in Fig. 5A, for 10 sequences selected by GMM in Dataset D, RaptScore demonstrated a strong correlation with binding activity ($R^2$ = 0.93) using the best-scoring definition. When this scoring definition was applied to BO-generated sequences, RaptScore maintained a high correlation ($R^2$ = 0.92). Notably, four of the top five sequences based on binding activity were also ranked among the top five sequences by RaptScore, confirming its utility in prioritizing high-binding candidates. Further analysis of sequences generated by L-models revealed that L-25 exhibited a strong correlation, whereas L-20 showed a weaker relationship. Importantly, for the L-20 model, although the correlation coefficient was low, four of the top five sequences in terms of binding activity were included among the top five ranked by RaptScore, reinforcing its ability to prioritize sequences (Supplementary Table S18). When extended to Dataset E, the correlation trends differed. As shown in Fig. 5B, RaptScore exhibited a moderate correlation with binding activity ($R^2$ = 0.52) for 10 sequences selected by GMM. Similarly, for BO-generated sequences, the correlation was $R^2$ = 0.47. Despite this lower correlation, RaptScore still successfully ranked four of the top five high-binding sequences, demonstrating its robustness across datasets. In the L-model analysis, the L-30 model exhibited a strong correlation, whereas L-35 showed a weaker correlation. Notably, for L-30, RaptScore correctly assigned higher scores to sequences with the highest binding activity, reinforcing its consistency (Supplementary Table S19). These findings suggest that integrating RaptScore with RaptGen can efficiently filter generated sequences, reducing the number of candidates requiring experimental binding activity measurements while still identifying high-binding sequences. Given that RaptScore consistently ranked top-performing sequences across multiple datasets, this approach could serve as a reliable screening tool, allowing experimental validation to focus on sequences with better RaptScores. By prioritizing these high-activity candidates, researchers can optimize resource allocation and reduce experimental workload, improving the overall efficiency of aptamer discovery.

## Discussion

This study introduces RaptScore, a novel computational approach leveraging LLMs to evaluate the binding activity of RNA aptamers beyond the constraints of traditional methods. Unlike existing metrics such as frequency and enrichment, which are limited to sequences present within SELEX rounds, RaptScore enables the assessment of arbitrary sequences, including those of varying lengths. This adaptability unlocks a more comprehensive exploration of aptamer space, facilitating not only sequence ranking, but also optimization through integration with *in silico* maturation methods. Such methods include GA and aptamer generation models like RaptGen, which can generate novel candidates but do not necessarily provide predictive information about the binding activity of arbitrary sequences. In this way, RaptScore privides a powerful framework for both sequence selection and truncation, opening the way to *in silico* aptamer evolution.

Experimental results collectively demonstrate the versatility of RaptScore across three key applications: ranking, sequence truncation, and integration with generative models. Regarding ranking, although RaptScore extends evaluation to arbitrary sequences beyond those observed in SELEX, it nevertheless exhibits a strong correlation with binding activity measured through SPR assays and achieves performance comparable to existing methods in identifying top-binding sequences, under the best-performing scoring settings. While there is variability across SELEX rounds (Fig. 2D–F and Table 1), this is an expected outcome because in later rounds tend to be progressively enriched with strong binders, enabling RaptScore to better capture the sequence patterns of high-activity aptamers. Furthermore, RaptScore facilitates the identification of truncated aptamers, a capability not addressed by conventional methods. Two scales of truncation were systematically evaluated: small-scale truncations of up to three nucleotides, and larger-scale truncations of 10 nucleotides. In both cases, truncated variants with comparable or improved binding activity relative to the parent aptamers were identified. Finally, the successful integration of RaptScore with RaptGen further underscores its utility, enabling efficient sequence exploration with reduced experimental effort. Notably, even in cases of moderate correlation like Dataset E (Fig. 5B), RaptScore functioned as an effective filter, identifying most of the best binders from only half of the candidates. Together, these findings highlight RaptScore’s potential to streamline computational and experimental workflows in aptamer research.

A key advantage of RaptScore is its ability to assess sequences independently of SELEX occurrence, thereby broadening the scope of aptamer discovery. However, this flexibility introduces certain challenges, particularly in parameter selection. The effectiveness of RaptScore is influenced by several factors, including the choice of SELEX round used for pretraining, how duplicate sequences are handled, and whether aptamer adapters are included, as shown in Fig. 2D–F. For instance, while pooling data from all rounds provided better performance than using early-round data, our analysis (Fig. 2D–F) revealed that the single later round was the best overall. This suggests that the benefits of pooling, such as the stabilizing effect of increased sample diversity, may be partially offset by the dilution of evolutionary signals and round-specific characteristics. Given that these parameters significantly impact correlation, careful parameter adjustment is essential for optimizing RaptScore’s performance. To address this, we conducted comparative analyses of the correlation between RaptScore and binding activity under various parameter settings. Nonetheless, further refinement of these settings warrants additional investigation, particularly regarding their application to diverse SELEX datasets and the challenge of integrating multi-round information without losing evolutionary signals.

Another limitation of RaptScore is its reliance solely on sequence-based evaluation, which does not explicitly account for secondary or tertiary structural features essential for aptamer- target interactions. Although enriched sequence motifs identified through SELEX generally correlate with high binding activity, structural factors significantly influence the actual aptamer function. This sequence-only approach can lead to two potential issues. First, this sequence-only approach may produce false negatives, failing to identify potent aptamers whose enrichment in SELEX is hindered by experimental biases introduced during PCR, transcription, and reverse transcription. For example, highly structured aptamers, which can be strong binders, may amplify inefficiently during PCR, leading RaptScore to assign them undeservedly low scores. Second, this limitation may also lead to false positives when evaluating novel or truncated variants. Indeed, using RaptScore as a guiding metric in GA-based truncation lead to extremely short aptamers with random regions as short as ~10 nucleotides as shown in Fig. 4B. Such short sequences, while achieving high scores in a purely sequence-based evaluation, may lack the necessary structural conformations required for target binding. Therefore, future work should aim to integrate structural modeling techniques, such as RNA secondary structure, to complement and enhance the sequence-based evaluations performed by RaptScore. In addition, other possible directions include the use of single-nucleotide or variable *k*-mer embeddings.

Despite these limitations, RaptScore represents a significant advancement in computational aptamer evaluation. By providing an efficient method for binding activity estimation, it reduces the reliance on exhaustive experimental screening while enabling the discovery of high-performance sequences. The successful application of RaptScore to aptamer truncation highlights its potential to optimize sequence length, which is essential for minimizing production costs and improving performance and reliability in industrial-scale manufacturing. Furthermore, the successful integration of RaptScore with RaptGen suggests that it could also be effectively combined with other aptamer generation methods, such as other deep learning-based aptamer generation models. This potential for broader applicability highlights RaptScore’s ability to enhance aptamer generation workflows, offering a pathway toward more efficient aptamer discovery and optimization.

In conclusion, RaptScore provides a versatile framework for evaluating, optimizing, and truncating RNA aptamers, addressing key limitations of conventional aptamer binding metrics. Its ability to assess sequences beyond SELEX data and integrate into computational optimization pipelines positions it as a valuable tool in RNA-based therapeutic and diagnostic development.

## Supplementary Material

gkaf1480_Supplemental_File

## Data Availability

An implementation of RaptScore is available on GitHub (https://github.com/hmdlab/RaptScore) and Zenodo (https://zenodo.org/records/17823406). The three newly acquired HT-SELEX Datasets A, B, and C are available as DRA019577, DRA019609, and DRA019610 in DDBJ. Also, Datasets D and E are available as DRA009383 and DRA009384.
