# Supplementary material for: RaptScore: a large language model-based algorithm for versatile aptamer evaluation
Source: Nucleic Acids Res. 2026 Jan 14;54(2):gkaf1480. doi: 10.1093/nar/gkaf1480 (PMC12802916; doi:10.1093/nar/gkaf1480)
Supplement: gkaf1480_Supplemental_File [file gkaf1480_supplemental_file.pdf]

Supplementary of "RaptScore: a large language model-based algorithm for versatile aptamer evaluation"

Akira Kimura-Yamazaki[1], Tatsuo Adachi[2], Shigetaka Nakamura[2], Yoshikazu Nakamura[2], and Michiaki Hamada*[1,3,4]

[1]Graduate School of Advanced Science and Engineering, Waseda University, Shinjuku-ku Okubo 3-4-1, 169-0072, Tokyo, Japan
[2]RIBOMIC, Minato-ku Shirokanedai 3-16-13, 108-0071, Tokyo, Japan
[3]Cellular and Molecular Biotechnology Research Institute (CMB), National Institute of Advanced Industrial Science and Technology (AIST), Koto-ku Aomi 2-3-26, 135-0064, Tokyo, Japan
[4]Graduate School of Medicine, Nippon Medical School, Bunkyo-ku Sendagi 1-1-5, 113-8602, Tokyo, Japan

1 Abbreviation

- BO: Bayesian Optimization
- BERT: Bidirectional Encoder Representations from Transformers
- GA: Genetic Algorithm
- LLM: Large Language Model
- SELEX: Systematic Evolution of Ligands by Exponential Enrichment
- VAE: Variational Auto-Encoder

2 Supplementary Text

2.1 Calculation of Frequency and Enrichment

2.1.1 Frequency Calculation

For each unique sequence $s \in S$, frequency is determined in each round by computing the ratio of its occurrences to the total occurrences of all unique sequences in that round. The frequency of a given sequence s at round x is calculated as:

$$\text{Frequency}_x(s) = \frac{\text{count}_x(s)}{\sum_{s' \in S} \text{count}_x(s')}$$

where $\text{count}_x(s)$ represents the number of times sequence s appears in round x.

2.1.2 Enrichment Calculation

The enrichment value for each unique sequence s across rounds is computed as the ratio of its frequency in the current round to that in the previous round. The enrichment score for sequence s in round x is given by:

$$\text{Enrichment}_x(s) = \frac{\text{Frequency}_x(s)}{\text{Frequency}_{x-1}(s)}$$

provided that $\text{Frequency}_{x-1}(s) \neq 0$ and that round x is not the initial round. If either of these conditions is not met, the enrichment score remains undefined.

*To whom correspondence should be addressed. Email: mhamada@waseda.jp

2.2 SELEX protocol

2.2.1 Dataset A

A single-stranded DNA (ssDNA) library, 5′-TACGCCTGCGTAGCTCCT-35N-AGCTCGACGGAG CTTCCCTATAGTGAGTCGTATTA-3′, was used as the template. Here 35N represents the 35-nt random sequence, and the underlined sequence indicates the complementary sequence of the T7 promoter. First, the ssDNA template was hybridized with the forward primer, 5′- TAATACGACTCACTATA GGGAAGCTCCGTCGAGCT-3′, and dsDNA was produced by DNA polymerase. The RNA pool was transcribed using T7 RNA polymerase containing Y639F mutation, GTP, ATP, dTTP, and dCTP. The resulting oligonucleotide pool was used for selection to the target protein (FGF-9, 273-F9, R&D systems), which was immobilized to NHS-activated Sepharose beads (17-0906-01, Cytiva). The selection was oligonucleotide pool and target protein were incubated in buffer A consisting of 145 mM NaCl, 5.4 mM KCl, 0.8 mM $MgCl_2$, 1.8 mM $CaCl_2$, 0.05% Tween20 and 20 mM Tris–HCl (pH 7.6). After incubation, the beads were washed three times with buffer, and RNA molecules bind to target protein eluted with 6M urea. To increase the stringency of the selection, the conditions were modified in each round, and the details of these conditions are summarized in the table. For the subsequent round of the selection and amplification, the pool was reverse transcribed by SuperScript IV (18090050, Thermo Fisher Scientific) and amplified by PCR. The dsDNA was used as the library for the next round.

Supplementary Table 1: DatasetA

Round	Contact					Wash		
	Protein	RNA	Buffer	Volume	Temp, Time	Buffer	Volume	Times
1	5 μg	10 μg	A	50 μL	25°C, 30 min	A	50 μL	3
2	5 μg	10 μg	A	100 μL	25°C, 30 min	A	100 μL	5
3	2.5 μg	5 μg	A	100 μL	25°C, 30 min	A	120 μL	5
4	1 μg	1 μg	A	100 μL	37°C, 10 min	A	150 μL	5
5	0.5 μg	1 μg	A	100 μL	37°C, 5 min	A	150 μL	5
6	0.5 μg	1 μg	A	100 μL	37°C, 5 min	A	150 μL	5

2.2.2 Dataset B

A single-stranded DNA (ssDNA) library, 5′-CGTGCAGAGATCCTC-35N-AGTCGAAGTTCTCCCT ATAGTGAGTCGTATTA-3′, was used as the template. the ssDNA template was hybridized with the forward primer, 5′- TAATACGACTCACTATAGGGAGAACTTCGACT-3′, and blunt end dsDNA was produced by DNA polymerase. The library was transcribed using T7 RNA polymerase containing Y639F mutation and nucleotides of GTP, ATP, dTTP, and 2'-F-CTP. The resulting oligonucleotide pool was used for selection to the target protein (ST2/IL-33R, 1004-MR, R&D systems). The subsequent procedures were carried out in the same manner as Dataset A. The conditions for each round are summarized in the table. Buffer B indicates Buffer A + 150 mM NaCl.

Supplementary Table 2: DatasetB

Round	Contact					Wash		
	Protein	RNA	Buffer	Volume	Temp, Time	Buffer	Volume	Times
1	5 μg	10 μg	A	100 μL	25°C, 30 min	A	100 μL	3
2	3 μg	10 μg	A	100 μL	25°C, 30 min	A	100 μL	3
3	2 μg	10 μg	A	100 μL	25°C, 20 min	B	200 μL	3
4	1 μg	10 μg	A	100 μL	25°C, 10 min	B	200 μL	5
5	1 μg	10 μg	A	100 μL	37°C, 3 min	B	200 μL	5
6	1 μg	5 μg	A	100 μL	37°C, 3 min	B	200 μL	5
7	1 μg	5 μg	A	100 μL	37°C, 3 min	B	200 μL	5

2.2.3 Dataset C

A single-stranded DNA (ssDNA) library, 5′-TCGAG-30N-TCCCTATAGTGAGTCGTATTA-3′, was used as template. The ssDNA template was hybridizaed with the forward primer, 5′-TACGAGGTAGC

ATGATAATACGACTCACTATAGGGA-3′, and the dsDNA was produced by DNA polymerase. The random library was transcribed using T7 RNA polymerase and nucleotides of ATP, GTP, dTTP, dCTP, and 10 molar excess condition of GMP relative to GTP. GMP was added to generate a monophosphorylated 5′ terminal, which is essential for following the ligation reaction. The target protein, selection, washing, and sequencing methods were carried out in the same manner as Dataset A. After collecting protein-bound oligonucleotides, the T7 promoter sequence 5′-TAATACGACTCACTATA-3′ was ligated to the 5′ terminus of the RNA in the presence of the forward bridge sequence 5′-ACCCTATAGTGAGTCGTATTA-NH_2-3′, and the 3′ terminus of the RNA was ligated to the reverse adaptor sequence 5′-p-GAAAAAATACAAAGCGTA-NH_2-3′ in the presence of the reverse primer sequence 5′-TACGCTTTGTATTTTTTCTCGAG-3′ by using T4 RNA ligase 2 (M0239, New England Biolabs), and reverse-transcribed by SuperScript IV. After PCR amplification, the dsDNA was digested by the XhoI restriction enzyme (R0146S, NEB) and used as the library for the next round. The conditions for each round are summarized in the table.

Supplementary Table 3: DatasetC

Round	Contact					Wash		
	Protein	RNA	Buffer	Volume	Temp, Time	Buffer	Volume	Times
1	2 μg	10 μg	A	50 μL	25°C, 30 min	A	100 μL	3
2	2 μg	10 μg	A	100 μL	25°C, 30 min	A	100 μL	5
3	1 μg	5 μg	A	100 μL	37°C, 20 min	A	100 μL	5
4	0.5 μg	2 μg	A	100 μL	37°C, 10 min	B	100 μL	5
5	0.5 μg	1 μg	A	100 μL	37°C, 3 min	B	100 μL	5
6	0.5 μg	0.5 μg	A	100 μL	37°C, 3 min	B	150 μL	5

2.3 Sequencing procedures

After the SELEX, the selected library was analyzed using a next-generation sequencing approach. A barcode sequence 5′-CCATCTCATCCCTGCGTGTCTCCGACTCAG-XXXXXXXXXX-GAT-3′ and a trP1 sequence 5′-CCTCTCTATGGGCAGTCGGTGAT-3′ were attached to the 5′ and 3′ ends of the library, respectively. Sequencing was performed using an Ion PGM instrument with the Ion PGM Hi-Q View Sequencing Kit (A30044, Thermo Fisher Scientific).

3 Supplementary Tables

Supplementary Table 4: The sequences selected from each round of Dataset A based on Frequency and Enrichment. RaptScore and Relative activities are shown. Relative activity is defined as the binding activity of each sequence scaled relative to the highest binding sequence selected by Frequency or Enrichment, which is set to 100. The sequences correspond to those presented in Figure 2A.

ID	Sequences	Relative Activity	RaptScore
Round1_1	ACATAGAGACTGAGGGTTAAACTAAGGCAGAGCAA	3.32	-138.9
Round1_2	GTAATGTTTTACCGGAACCGAGGCGAGCGGTAACG	-1.26	-130.74
Round1_3	TCGCCGGGTCACAGGGATTATGAACCCCATCATAC	2.93	-118.87
Round1_4	TTGACCTAAGGCAAGGTTAATAGGGGTAGGACCTC	-0.31	-112.27
Round1_5	CCTCACATTTGTCGCAGTGTGGTGGTAGCGTCTGA	-1.33	-122.36
Round1_6	GTGATTAATCATAGATAGTTAATCTGTCGACATAC	-0.98	-138.77
Round2_1	ACAGCGTCACGCAACAATACGAAAACTGTTCGGCC	-2.05	-131.65
Round2_2	GGGTCTCGCCATAACACGACACTGTAGTAATATGT	-1.17	-122.47
Round2_3	TTTCTGTCCAGTGTGAAGCTGCGAAAACCTCACCC	-2.03	-124.07
Round2_4	ATGCATTGTCGCAGAAAGATCCGGTTTAGCCGAAG	-0.07	-118.54
Round2_5	CTAGATGAGGTGAATGCGTCACAACGCTTATGCAA	-1.42	-129.62
Round2_6	AGAAGCGAATCTGTCGCACAATAAAGTTCTGTAGT	0.0	-121.69
Round3_1	GCTAAACAAAGCGACCGCTGGGTCACCAATGAAAG	18.37	-125.28
Round3_2	TGGACCAGCCCATAGATATATTCATCGGCTTGCTA	1.93	-115.63
Round3_3	ACTTCAAGGAAACTTTGTTCCACCTAACAGCAGGG	0.63	-132.94
Round3_4	TCTTGACAAGGCTGCCGTCACAAAGGGAAGACATT	27.32	-89.8
Round3_5	TGGACCTGACGAATAGTGATTCTGCGCATTCTTGA	8.08	-120.07
Round3_6	AGCGATGTTGCATCGCAAGAAATCAAATGAGTCCC	19.26	-120.85
Round4_1	TAAATTCGATATCGTACACGCCAAAGCTTGTGCTA	24.82	-122.86
Round4_2	GCTACAAGGTTGCCGTAGCCCCAATCAAGCTTATC	37.44	-81.45
Round4_3	GGCGCGGTGTTGAGCGCAAACTAACTCATTTCGTC	36.8	-119.26
Round4_4	GACAAGGGAGTCAAGGTTGCCGACTCCGAACCAGC	63.21	-86.11
Round4_5	CGAAAAATGACGGTGTAGAGTCATAATTTCGATGG	100.0	-105.36
Round4_6	TGGACCGCTGACAGCGCACGAATTAAAACCTCAGC	6.34	-114.2
Round5_1	TGCCACACAACCAAGGTTGCCGGTTGTGTTATCAA	95.57	-66.69
Round5_2	ATGAGTGGGCCGGTGTAGAGGCCCACAACCATGTG	84.35	-96.95
Round5_3	TGTATAATCCGGACGGTCAAGGCTGCCGACCGACA	53.1	-79.14
Round5_4	TGGACCAGTGACACTGTGAAGTGTAGTTTGGACAT	61.45	-105.5
Round5_5	AAACAGGATCCCCAAGGTTGCCGGGGTTCCAATCA	55.0	-68.09
Round5_6	TGGACCGAATGTGAGGGTGCCCACGTGCCAACCGA	30.14	-103.03
Round6_1	AATTGATGCAACTTATAGTTGGCACCGACCTGCAC	48.24	-127.99
Round6_2	TAACTGGGGGCATCTCGGTTGCCGGGCCCTAACCA	46.37	-102.38
Round6_3	GCTCGACTTTAGTGATTCGTAGCTTCTAATTACCC	15.99	-130.46
Round6_4	GAGGGCACCCAAGGCTGCCGGGTGCCATGCACACA	56.09	-71.33
Round6_5	CACCAGCGCGAATTCGGATTGCCGACGCGCGACTC	94.02	-108.24
Round6_6	GCCTACGAAGTCCAAGGCTGCCGGACTTGATTTCC	22.28	-81.08
Random control	NNNNNNNNNNNNNNNNNNNNNNNNNNNNNNNNNNN	-3.87	-

Supplementary Table 5: The sequences selected from each round of Dataset B based on Frequency and Enrichment. RaptScore and Relative activities are shown. Relative activity is defined as the binding activity of each sequence scaled relative to the highest binding sequence selected by Frequency or Enrichment, which is set to 100. The sequences correspond to those presented in Figure 2B.

ID	Sequences	Relative Activity	RaptScore
Round0_1	TGTGAGGCCCTGACCATTTGCAGTCTAACACTACC	-6.37	-45.36
Round0_2	ATGACCTCCGAACTCCTCTCGCTATTCGAAAGTTA	-3.59	-40.27
Round0_3	TCCTGGCCATTTTTGGTGTTTTCCCTTGAATACAG	-5.79	-45.20
Round0_4	CGGTTCCATCACAGGAGCCTTTACACCTCCGCAGA	-3.87	-45.44
Round0_5	GCATTTAACGATTATCCCGGGCCTCAGCCGGCGTG	-2.20	-47.80
Round0_6	TTTAAGTTGAACCCCGCTTACCTTTGTCTCCCTCA	-3.31	-43.34
Round1_1	TCGCGAACGCTTAAAGAGTCTTATGACGTAATTGT	-3.30	-36.07
Round1_2	AATCGCCTGCGCGTAAGTATATTAATTGCTAACAA	-6.62	-41.02
Round1_3	ATAACGTAAAGAGTCCATAAAGGGACGTAATTGTT	-4.69	-45.01
Round1_4	GTCGTAAAGAGCCGAAAATAGGCGTAATTGTTCCA	-6.89	-40.79
Round1_5	ATGCAAATAACTCCCTGAGTAATACATGACATGGT	-9.10	-42.17
Round1_6	ACCCTAGAGCGCGTGAATATATTAATAGCTAACAA	-3.31	-45.41
Round2_1	TCGCGAACGCTTAAAGAGTCTTATGACGTGATTGT	-3.31	-36.67
Round2_2	TCGCGAACGCTTAACGAGTCTTATGACGTAATTGT	-3.85	-37.30
Round2_3	TCGCGAACGCTTAAAGAGTCTTATGGCGTAATTGT	-5.79	-37.55
Round2_4	AAAGAGCCTGTTAAAGGCGCAATTGTTATATAACT	7.15	-43.57
Round2_5	TATAACGTTAAATACGTTTAAAACCCATTGGTCAA	18.97	-43.97
Round2_6	AAAGAGCCAGAAGGCATAATTGTTTACATTAAACT	22.03	-44.79
Round3_1	GTCAACCTTTCAGCGACAGCCGATTCTGAATAGTA	39.70	-43.97
Round3_2	GCTTGGTAATATGCGCGAAGAACCTCCAACATTTA	56.11	-16.66
Round3_3	TGGCGTGTAGGCCTTTGACCTCCTTATACGCCATT	27.90	-40.02
Round3_4	CCACGTGGTCAATCACCGATGTGACACCATTCAAA	73.58	-28.06
Round3_5	GTCGTAAAGAGCCGAAAATAGGCGTAATTGTTCCT	3.86	-40.08
Round3_6	TCGCGAACGCTTAAAGAGTCTTATGATGTAATTGT	36.29	-34.84
Round4_1	CCAAAACAACGCGTACGATGTGTACTCCGACGCTA	84.73	-23.04
Round4_2	GCTTGATAATATGCGCGAACCTCCAAACGATACTA	84.14	-16.53
Round4_3	CCCCAATCCGCATGCGTACGATGCGTACTCCGACG	77.58	-24.15
Round4_4	GCTTGGTAATATGCGCGAATTATCTCTCGGATGAA	72.23	-20.84
Round4_5	ACCCCGCGGCGCGTACGATGAGTACTCCGACACGC	84.42	-23.20
Round4_6	TCTGCGAGAAGATCCAGAAGCCTTACGATCTGATT	65.76	-28.45
Round5_1	CAAACGGACGTGCATGCTTCGTGCCCGATGTCAAC	16.72	-34.73
Round5_2	ACCATTATTGGTTTGTACCCCAGTCACTTCTCCAT	41.51	-28.41
Round5_3	ACACGGACCGATGTCCTACGATCCTCTCCTGAACA	82.50	-27.06
Round5_4	TGTAACAAGCTGTTTTGGAACAGCGCATAACGATA	59.41	-31.36
Round5_5	ATGCCAATACACGCACCGATGTGCAAGGCAAAATC	98.59	-22.45
Round5_6	CTTGTGCACACTTTACGACGAGTACTCCGGAAGTG	67.45	-24.33
Round6_1	TTCAGCGCCAATCACCGATGTGACCCGCTGTAAAT	95.59	-36.83
Round6_2	ACACGGACCGATGTCCTACGTAAGTCTCTAGAGAT	84.67	-31.99
Round6_3	CGCCGGGACCGGCCAACACAGGCCTGATGTCAACC	25.68	-40.91
Round6_4	ACACGGACCGATGTCCTACGATCCTCTCCCGAACA	78.58	-26.28
Round6_5	ACACGCACACCGACAACAAAGTCGTTGATGTCAAG	44.70	-34.23
Round6_6	ACGATGCGTACTCCGAGTAAAGTTCTATCTGGGCA	73.21	-24.46
Round7_1	CGCACGGACACGCGGAATTTTCGCTTGATGTCAAC	51.38	-32.44
Round7_2	CGCACGGACACGCGGTAATTTCGCTTGATGTCAAC	41.73	-29.94
Round7_3	CCAACTCCACGTATTTCGAACGACCTCGAGAAATC	100.00	-37.30
Round7_4	CGCACGGACACGCGGCATTTTCGCTTGATGTCAAC	24.82	-32.82
Round7_5	CGCCGAGACACGAAATGAAGAACGTGATGTCAACT	43.83	-32.92
Round7_6	GCCGGGACAGTAGTGAAAAACCTCTGATGTCAACC	43.57	-33.38
Random control	NNNNNNNNNNNNNNNNNNNNNNNNNNNNNNNNNNN	-1.34	-

Supplementary Table 6: The sequences selected from each round of Dataset C based on Frequency and Enrichment. RaptScore and Relative activities are shown. Relative activity is defined as the binding activity of each sequence scaled relative to the highest binding sequence selected by Frequency or Enrichment, which is set to 100. The sequences correspond to those presented in Figure 2C.

ID	Sequences	Relative Activity	RaptScore
Round2_1	GAGACCAAGGCTGCCGGTCTATATAGCAAG	6.85	-194.48
Round2_2	GGAGGTCCAAGGCTGCCGGACAACATGTAG	4.26	-224.10
Round2_3	AATGGTCCCATACCACCACTTATAAAGTAG	-2.06	-301.85
Round2_4	AGATTAGTCCATGATTAGCAGTGTAGCCGG	-1.35	-297.87
Round2_5	AGTAGATGTCCCAAAGCTACTTGTTGCGAT	-2.01	-297.81
Round2_6	AAAGCCATTAGCTGCTGGTGACATAAATTA	-0.27	-301.67
Round3_1	GAAAGGGATGTGCCGCCTCACGCGTGACCG	-0.24	-259.83
Round3_2	GAGGCCAAGGTTGCCGGTCTCGGATATGAG	80.83	-200.19
Round3_3	GATGGTACAAGGTTGCCGTACCAACACTGG	73.62	-220.46
Round3_4	AAGAAGTTCCTTAATAACTAGTACAAATAG	-1.83	-308.22
Round3_5	AGATAGTCCACCCGGCCGGTTTGCAGGCTG	-1.07	-277.47
Round3_6	GAAGGAAACGTGTTCCTGACTAATGAATGG	-0.99	-278.16
Round4_1	AGGGGTTGCCCACGTACAAACCACAACTAG	-1.46	-261.89
Round4_2	GAAAGGGTAGCCGCCCCACGCGTGACCGAA	3.04	-247.98
Round4_3	GAAGGATGAAGGACCCGTCACTGTGAAGTG	3.74	-285.87
Round4_4	GAGTAATATTGTACGTCCGGCCAAGAGCTG	-1.52	-286.02
Round4_5	GGAGATACCAAGGTTGCCGGTATCACTAGG	100.00	-212.62
Round4_6	AGGACCAAGGTTGCCGGTCCACGGTACTAG	45.02	-187.15
Round5_1	AGAGGCAAGGTTGCCGCCTCATGTGAAGTG	44.24	-209.44
Round5_2	GGAGACCAAGGCTGCCGGTCATCATACATG	6.45	-205.51
Round5_3	GGATGCAAGGTTGCCGCATCATATAGGAAG	80.25	-214.65
Round5_4	GACAGCCAAGGTTGCCGGATTGTCCAACAG	46.06	-230.08
Round5_5	GGAGACCAAGGCTGCCGGTCATCATGCATG	5.72	-213.03
Round5_6	GGATCCAAGGTTGCCGGATCGCACAGGTAG	78.84	-218.38
Round6_1	GAGACCAAGGCTGCCGGTCTTTATAGCAAG	24.43	-204.98
Round6_2	ATAGGTTGCCGCCCCTGGATAAAAGAGGTG	48.17	-210.47
Round6_3	GACAGCCAAGGTCGCCGGATTGTCCAACAG	4.48	-264.53
Round6_4	GAGTGCCAAGGCTGCCGGCACTGATTATAG	22.35	-207.07
Round6_5	GTAGACCAAGGTTGCCGGTCACACAATATG	86.93	-187.85
Round6_6	GGAGACCAAGGCTGCCGGTCTTACACGTAG	19.75	-211.89
Random control	NNNNNNNNNNNNNNNNNNNNNNNNNNNNNN	-0.10	-

Supplementary Table 7: The sequences selected from each round of Dataset A based on RaptScore. RaptScore and Relative activities are shown. Relative activity is defined as the binding activity of each sequence scaled relative to the highest binding sequence selected by Frequency or Enrichment, which is set to 100. The sequences correspond to those presented in Table1 A.

ID	Sequences	Relative Activity	RaptScore
Round1_1	ACAACGTGTGACGTGTGGCATGTATGCATACACGG	7.78	-99.28
Round1_2	AAAGGTCAAACAGAAAAAAAAAGGTCAAACTAATG	4.68	-101.12
Round1_3	CAACACACCCGGCACAAAACGATAAAACGATAACC	-2.41	-101.47
Round2_1	TAACAGTTAAGCCCTGCGATGAATACGATGAATAT	18.08	-102.01
Round2_2	TAGATGTAAACCATGACAATGTATGCATTGTATAT	8.64	-103.01
Round2_3	CAATGCATGTGAATGCAGTGATTTTGTAATGCAGT	99.6	-103.31
Round3_1	TGGACTGGACTGGATTGCATGTACAGAATCATCGA	60.71	-94.64
Round3_2	TGAATGTGAAAAAGAGCAAATGCAAATGTGAATTT	22.35	-96.02
Round3_3	TGGACTGAGCACTTGTGTGAGCAAAATAAAAAGCG	75.89	-97.47
Round4_1	ATTGTGCTGAAGCCCGAGGTTGCCGGGTTGCAACT	31.33	-71.83
Round4_2	GTGACACAACCCAGGTTGCCAGGTTGCCCATATAT	19.13	-72.02
Round4_3	GTCTAAAAGATTGCCAAGGTTGCCGGATCAAAAAG	40.41	-72.23
Round5_1	GTTCGCCAACCAAGGTTGCCGGTTGCCGCGCCCTT	29.77	-49.54
Round5_2	GCTACCAAGGCTGCAAGGTTGCCGCAGCCAATATA	83.38	-50.57
Round5_3	AGCCAAGGTTGAGGTATCCAAGGTTGCCGGGTACA	76.18	-50.68
Round6_1	GATCCCAAGACTGCCCAAGGTTGCCGGGTAGTTTA	92.72	-50.39
Round6_2	GCTACCAAGGCTGCAAGGTTGCCGCAGCCAATATA	90.18	-50.57
Round6_3	AGCACAAGGGTGCCAAGGTTGCCGGCACTGAACAC	77.54	-51.45

Supplementary Table 8: The sequences selected from each round of Dataset B based on RaptScore. RaptScore and Relative activities are shown. Relative activity is defined as the binding activity of each sequence scaled relative to the highest binding sequence selected by Frequency or Enrichment, which is set to 100. The sequences correspond to those presented in Table1 B.

ID	Sequences	Relative Activity	RaptScore
round0_1	CTCTCCCAACCCTCCCCAACCCTACCCCACCCACG	-1.21	-33.20
round0_2	AAACAACCACCCCACCCCCCACACCAAAAGAATAC	-3.20	-34.03
round0_3	CTATCCCCACCCCTTCCCCAAATACCAAAACCCGG	-1.60	-34.76
round1_1	GAAAAATAAAATAAGACATAAAAATAATAATCCCT	-6.03	-34.46
round1_2	TTCCCGCCCCAAAGAAGACGTGCAGAGACCTCCAG	-5.21	-34.73
round1_3	AAAACAAATAAAATGAAACTGCAAACAAAATCGCT	-4.82	-36.60
round2_1	GTGCAAAAGCGAGTCTCGCCAAAAAAAAAAAAAAC	-4.45	-33.48
round2_2	CTCCATATAATAAATATCTCTATATCTCTATACCT	-6.85	-34.32
round2_3	CGATCTTCCCTAGACGCGAAAAAAAAAAAAAACAT	-6.05	-34.68
round3_1	GCCTATGTCAGCCTATTGAAGTATCTCAACATTAT	26.33	-16.13
round3_2	GCCTATGTCAGCCTTACGCATATCTCCAGCCTAAA	-5.70	-16.48
round3_3	GCCAAATGTCAGCCTATGTAAGTATCTCGTCTAAT	-1.22	-17.54
round4_1	GCTTGATAATATGCGCGCAAGTACTCACACGTCCT	-0.41	-15.11
round4_2	GCTTGATAATATGCGCGCAAGTACTCACACGTCTT	10.60	-15.34
round4_3	GCTTGATAATATGCGCGAAGAACCTCCAACATTTA	54.90	-15.86
round5_1	GCTTGATAATATGCGCGAACCTCCAAACGATGCTA	81.20	-13.04
round5_2	GCTTGATAATATGCGCGAAAGTCCCTTAACAAACA	68.59	-13.97
round5_3	GCTTGATAATATGCGCGAACCTCCAAACGATACGA	87.42	-14.66
round6_1	TGTAACAAGCTGTTTTGGAACAGCGCATAACGATA	59.41	-0.56
round6_2	TGTAACAAGCTGTTTTGGAACAGCGCATAACGAAC	-4.91	-0.57
round6_3	TGTAACAAGCTGTTTTGGAACAGCGCATAACGAAA	-3.28	-0.58
round7_1	TGTAACAAGCTGTTTTGGAACAGCGCATAACGATC	-4.12	-0.51
round7_2	TGTAACAAGCTGTTTTGGAACAGCGCATAACGATA	59.41	-0.52
round7_3	TGTAACAAGCTGTTTTGGAACAGCGCATAACGATT	-0.82	-0.52

Supplementary Table 9: The sequences selected from each round of Dataset C based on RaptScore. RaptScore and Relative activities are shown. Relative activity is defined as the binding activity of each sequence scaled relative to the highest binding sequence selected by Frequency or Enrichment, which is set to 100. The sequences correspond to those presented in Table1 C.

ID	Sequences	Relative Activity	RaptScore
round2_1	AAGATTGTCCCCACCAAATAATAATGTGAG	0.89	-237.35
round2_2	AAAGTGTCCCTAATAATTATGTGTATATAG	-0.33	-239.16
round2_3	AAAATGTCCTATATCCATATATATAAGTGG	0.03	-239.67
round3_1	GAAAGGTTGCCGCCCCGCACACACAAACAG	34.32	-203.90
round3_2	AGGGTGTCCCCGCACAAATAATATATGTGG	0.84	-208.63
round3_3	AAAGGATTGCCGCCCGCCTACACATGCATG	32.22	-208.95
round4_1	GAGGCCAAGGTTGCCGGCCACACTAAACAC	25.90	-178.40
round4_2	AGGGCCAAGGTTGCCGGCCCACTAATAACT	57.52	-180.46
round4_3	GAAGCCAAGGTTGCCGGCCACAAAAATAGA	39.48	-181.05
round5_1	GAGGCCAAGGTTGCCGGCCCAATAAAAATG	61.55	-127.77
round5_2	GAGACCAAGGTTGCCGGTCTATATAGCAAG	50.17	-127.95
round5_3	TAGACCAAGGTTGCCGGTCTATATAGCAAG	101.17	-127.96
round6_1	TAGACCAAGGCTGCCGGTCTATATAGCAAG	20.70	-85.38
round6_2	AAGACCAAGGCTGCCGGTCTATATAGCAAG	5.87	-85.70
round6_3	GAGACCAAGGCTGCCGGTCTATATAGCAAA	5.62	-85.83

Supplementary Table 10: RaptScore-guided truncated aptamers from Dataset A. A 35-nt aptamer was truncated to generate aptamers with lengths ranging from 32 to 34 nucleotides. RaptScore and Relative activities are shown. Relative activity is defined as the binding activity scaled relative to the full-length aptamer. The sequences correspond to those presented in Figure 3A.

ID	Sequences	Length	Relative Activity	RaptScore
len_35_original	CGAAAAATGACGGTGTAGAGTC ATAATTTCGATGG	35	100.0	-
len_34_1	CGAAAATGACGGTGTAGAGTCA TAATTTCGATGG	34	87.47	-99.65
len_34_2	CGAAAAATGACGGTGTAGAGTC ATAATTCGATGG	34	92.7	-99.69
len_34_3	GAAAAATGACGGTGTAGAGTCA TAATTTCGATGG	34	79.98	-100.15
len_33_1	GAAAAATGACGGTGTAGAGTCA TAATTCGATGG	33	84.35	-93.24
len_33_2	CGAAAAATGACGGTGTAGAGTC ATAATCGATGG	33	93.3	-93.39
len_33_3	CGAAAAATACGGTGTAGAGTCA TAATTCGATGG	33	34.79	-93.64
len_32_1	CGAAAAATACGGTGTAGAGTCA TAATTCATGG	32	27.53	-88.00
len_32_2	CGAAAAATGACGGTGTAGAGTC ATAATTCGAT	32	91.64	-88.27
len_32_3	CGAAAAATACGGTGTAGAGTCA TAATTCGATG	32	33.62	-88.68

Supplementary Table 11: RaptScore-guided truncated aptamers from Dataset A. A 35-nt aptamer was truncated to generate aptamers with lengths ranging from 32 to 34 nucleotides. RaptScore and Relative activities are shown. Relative activity is defined as the binding activity scaled relative to the full-length aptamer. The sequences correspond to those presented in Figure 3B.

ID	Sequences	Length	Relative Activity	RaptScore
len_35_original	TGCCACACAACCAAGGTTGCCG GTTGTGTTATCAA	35	100.00	-
len_34_1	TGCCACACAACCAAGGTTGCCG GTTGTGTTACAA	34	112.00	-56.71
len_34_2	TGCCACACAACCAAGGTTGCCG GTTGTGTTATAA	34	107.65	-60.95
len_34_3	GCCACACAACCAAGGTTGCCGG TTGTGTTATCAA	34	90.29	-61.36
len_33_1	GCCACACAACCAAGGTTGCCGG TTGTGTTACAA	33	98.09	-52.01
len_33_2	TGCCACACAACCAAGGTTGCCG GTTGTGTTACA	33	115.25	-53.13
len_33_3	TGCACACAACCAAGGTTGCCGG TTGTGTTACAA	33	89.43	-53.38
len_32_1	GCCACACAACCAAGGTTGCCGG TTGTGTTACA	32	102.42	-48.10
len_32_2	TGCCAACACCAAGGTTGCCGGT TGTGTATCAA	32	28.42	-48.62
len_32_3	TGCCAAAACCAAGGTTGCCGGT TGTGTATCAA	32	55.12	-49.04

Supplementary Table 12: RaptScore-guided truncated aptamers from Dataset A. A 32-nt aptamer was further truncated to generate aptamers with lengths ranging from 29 to 31 nucleotides. RaptScore and Relative activities are shown. Relative activity is defined as the binding activity scaled relative to the full-length aptamer. The sequences correspond to those presented in Figure 3C.

ID	Sequences	Length	Relative Activity	RaptScore
len_35_original	TGCCACACAACCAAGGTTGCCGGT TGTGTTATCAA	35	100.00	-
len_31_high_1	GCCACACAACCAAGGTTGCCGGTT GTGTTAC	31	91.57	-45.45
len_31_high_2	GCCACACAACCAAGGTTGCCGGTT GTGTACA	31	100.95	-45.53
len_31_high_3	GCACACAACCAAGGTTGCCGGTTG TGTTACA	31	96.44	-46.72
len_31_low_1	GCCACACAACCAAGTTGCCGGTTG TGTTACA	31	47.15	-73.74
len_31_low_2	GCCACACAACCAAGGTTGCGGTTG TGTTACA	31	50.35	-77.32
len_31_low_3	GCCACACAACCAAGGTTCCGGTTG TGTTACA	31	29.03	-90.68
len_30_high_1	GCCACACAACCAAGGTTGCCGGTT GTGTAA	30	98.23	-41.70
len_30_high_2	GCCACACAACCAAGGTTGCCGGTT GTGTCA	30	97.88	-41.87
len_30_high_3	GCCACACAACCAAGGTTGCCGGTT GTGTTA	30	100.74	-41.90
len_30_low_1	GCCACACAACCAAGGTGCGGTTGT GTTACA	30	31.16	-94.48
len_30_low_2	GCCACACAACCAAGTTGCGGTTGT GTTACA	30	36.81	-96.11
len_30_low_3	GCCACACAACCAGGTTCCGGTTGT GTTACA	30	28.89	-101.50
len_29_high_1	GCCACACAACCAAGGTTGCCGGTT GTACA	29	78.99	-37.31
len_29_high_2	GCCACACAACCAAGGTTGCCGGTT GTGCA	29	103.12	-37.90
len_29_high_3	GCCACACCCAAGGTTGCCGGTTGT GTACA	29	32.69	-38.32
len_29_high_4	GCCACACAACCAAGGTTGCCGGTT GTGTC	29	100.83	-38.54
len_29_low_1	GCCACACAACCAAGGTTCCGTTTG TTACA	29	41.60	-103.45
len_29_low_2	GCCACACAACCAGGTTCCGGTTTG TTACA	29	32.12	-103.92
len_29_low_3	GCCACACAACCAAGGGCGGTTGT GTTACA	29	24.42	-104.28
len_29_low_4	GCCACACAACCAGGTTCCGGTTGT TTACA	29	23.41	-106.18

Supplementary Table 13: RaptScore-guided truncated aptamers from Dataset B. A 35-nt aptamer was truncated to generate aptamers with lengths ranging from 32 to 34 nucleotides. RaptScore and Relative activities are shown. Relative activity is defined as the binding activity scaled relative to the full-length aptamer. The sequences correspond to those presented in Figure 3D.

ID	Sequences	Length	Relative Activity	RaptScore
len_35_original	ATGCCAATACACGCACCGATGTGCAAGGCAAAATC	35	100.00	-
len_34_high_1	ATGCCAATACACGCACCGATGTGCAAGGCAAAAT	34	111.78	-22.34
len_34_high_2	ATGCCAATACACGCACCGATGTGCAAGGCAAAAC	34	114.91	-22.35
len_34_high_3	TGCCAATACACGCACCGATGTGCAAGGCAAAATC	34	110.79	-22.86
len_34_low_1	ATGCCAATACACGCACCGAGTGCAAGGCAAAATC	34	11.27	-31.19
len_34_low_2	ATGCCAATACACGCACCGTGTGCAAGGCAAAATC	34	1.96	-31.70
len_34_low_3	ATGCCAATACACGCACCATGTGCAAGGCAAAATC	34	18.12	-33.31
len_33_high_1	ATGCCAATAAGCACCGATGTGCAAGGCAAAATC	33	-2.88	-21.92
len_33_high_2	ATGCCAATACACGCACCGATGTGCAAGAAAATC	33	4.43	-22.17
len_33_high_3	ATGCCAATACACGCACCGATGTGCAGGAAAATC	33	0.63	-22.38
len_33_low_1	ATGCCAATACCGCACCGAGTGCAAGGCAAAATC	33	-6.20	-37.06
len_33_low_2	ATGCCAATACCGCACCGTGTGCAAGGCAAAATC	33	-5.53	-37.35
len_33_low_3	ATGCCAATACCGCACCATGTGCAAGGCAAAATC	33	-5.78	-38.65
len_32_high_1	ATGCCAATACACGCACCGATGTGCAGAAAATC	32	-1.81	-19.27
len_32_high_2	ATGCCAATACACGCACCGATGTGCAAAAAATC	32	0.90	-21.01
len_32_high_3	ATGCCAATACACGCACCGATGTGAAGAAAATC	32	-5.63	-21.29
len_32_low_1	ATGCCAATACCGCACCATGTGAAGGCAAAATC	32	-6.17	-39.51
len_32_low_2	ATGCCAATACCGCACCATGTGCAGGCAAAATC	32	-4.64	-39.76
len_32_low_3	ATGCCAATACCGCACCATGTGCAAGGAAAATC	32	-4.89	-39.99

Supplementary Table 14: RaptScore-guided truncated aptamers from Dataset B. A 35-nt aptamer was truncated to generate aptamers with lengths ranging from 32 to 34 nucleotides. RaptScore and Relative activities are shown. Relative activity is defined as the binding activity scaled relative to the full-length aptamer. The sequences correspond to those presented in Figure 3E.

ID	Sequences	Length	Relative Activity	RaptScore
len_35_original	CCAACTCCACGTATTTCGAACGAC CTCGAGAAATC	35	100.00	-
len_34_high_1	CCAACTCCACGTATTTCGAACACC TCGAGAAATC	34	7.28	-33.66
len_34_high_2	CCAACTCCACGTATTTCGAAGACC TCGAGAAATC	34	-4.73	-34.77
len_34_high_3	CCAACTCCACGTATTTCGAACGCC TCGAGAAATC	34	20.76	-35.56
len_34_low_1	CCAACTCCACGTATTTCGAACGAC CTCGGAAATC	34	-4.08	-38.53
len_34_low_2	CCAACTCCACTATTTCGAACGACC TCGAGAAATC	34	-3.13	-39.77
len_34_low_3	CCAACTCCCGTATTTCGAACGACC TCGAGAAATC	34	8.75	-40.01
len_33_high_1	CCAACTCCACGTATTTCGAACACC TCGAAAATC	33	-5.31	-32.66
len_33_high_2	CAACTCCACGTATTTCGAACACCT CGAGAAATC	33	6.54	-32.83
len_33_high_3	CCAACTCCACGTATTTCGAACACC TCGAGAAAC	33	-5.27	-32.96
len_33_low_1	CCAACTCCACTATTTCGAACGACC TCGGAAATC	33	-5.88	-40.79
len_33_low_2	CCAACTCCCGTATTTCGAACGACC CGAGAAATC	33	-3.70	-41.20
len_33_low_3	CCAACTCCCGTATTTCGAACGACC TCGGAAATC	33	-5.54	-41.21
len_32_high_1	CAACTCCACGTATTTCGAACACCT CGAAAATC	32	-5.22	-31.57
len_32_high_2	CCAACTCCACGTATTTCGAACACT CGAAAATC	32	-5.83	-31.61
len_32_high_3	CCAACTCCACGTATTTCGAACACC TCGAAAAC	32	-5.52	-31.72
len_32_low_1	CCAACTCCACTATTTGAACGACCC GAGAAATC	32	-5.80	-41.75
len_32_low_2	CCAACTCACGTATTTGAACGACCC GAGAAATC	32	-4.88	-41.98
len_32_low_3	CCAACTCCCGTATTTGAACGACCC GAGAAATC	32	-4.57	-42.26

Supplementary Table 15: RaptScore-guided truncated aptamers from Dataset B. A 35-nt aptamer was further to generate aptamers with lengths ranging from 32 to 33 nucleotides. Truncation position was decided based on the result of single nucleotide truncation shown in Figure 3D. RaptScore and Relative activities are shown. Relative activity is defined as the binding activity scaled relative to the full-length aptamer. The sequences correspond to those presented in Figure 3F.

ID	Sequences	Length	Relative Activity
len_35_original	ATGCCAATACACGCACCGATGTGCAAGGCAAAATC	35	100.00
len_33_high_1	ATGCCAATACACGCACCGATGTGCAAGGCAAAA	33	108.52
len_33_high_2	TGCCAATACACGCACCGATGTGCAAGGCAAAAT	33	107.47
len_33_high_3	TGCCAATACACGCACCGATGTGCAAGGCAAAAC	33	109.95
len_32	TGCCAATACACGCACCGATGTGCAAGGCAAAA	32	106.31

Supplementary Table 16: RaptScore-guided truncated aptamers from Dataset C. A 30-nt aptamer was truncated to generate aptamers with lengths ranging from 28 to 29 nucleotides. RaptScore and Relative activities are shown. Relative activity is defined as the binding activity scaled relative to the full-length aptamer. The sequences correspond to those presented in Figure 3G.

ID	Sequences	Length	Relative Activity	RaptScore
len_30_original	GGAGATACCAAGGTTGCCGGTATCACTAG G	30	100.00	-
len_29_high_1	GGAGATACCAAGGTTGCCGGTATACTAGG	29	107.10	-192.38
len_29_high_2	GGAGATACCAAGGTTGCCGGTACACTAGG	29	39.49	-193.23
len_29_high_3	GGAGATACCAAGGTTGCCGGTATCATAGG	29	117.51	-197.36
len_29_high_4	GGAGATACCAAGGTTGCCGGTATCACAGG	29	115.40	-198.36
len_29_high_5	GGAGATACCAAGGTTGCCGGTATCACTAG	29	62.90	-199.61
len_29_low_1	GGAGATCCAAGGTTGCCGGTATCACTAGG	29	9.65	-211.14
len_29_low_2	GGAGATACCAGGTTGCCGGTATCACTAGG	29	88.96	-213.45
len_29_low_3	GGAGATACCAAGTTGCCGGTATCACTAGG	29	13.96	-237.97
len_29_low_4	GGAGATACCAAGGTTGCGGTATCACTAGG	29	21.03	-241.61
len_29_low_5	GGAGATACCAAGGTTCCGGTATCACTAGG	29	6.40	-264.05
len_28_high_1	GGAGATACCAAGGTTGCCGGTATATAGG	28	58.98	-177.63
len_28_high_2	GGATACCAAGGTTGCCGGTATCACTAGG	28	119.28	-178.33
len_28_high_3	GGGTACCAAGGTTGCCGGTATCACTAGG	28	86.56	-178.64
len_28_high_4	GGAGACCAAGGTTGCCGGTATCACTAGG	28	5.25	-178.66
len_28_high_5	AGATACCAAGGTTGCCGGTATCACTAGG	28	102.20	-178.75
len_28_low_1	GGAGATACCAAGGTTCCGTATCACTAGG	28	1.65	-257.48
len_28_low_2	GGAGATACCAAGGTTCCGGTATCCTAGG	28	4.30	-259.86
len_28_low_3	GGAGATACCAAGGTTCCGGTTCACTAGG	28	48.64	-262.19
len_28_low_4	GGAGATACCAAGGTTCGGTATCACTAGG	28	0.98	-263.44
len_28_low_5	GGAGATACCAAGGTTGGGTATCACTAGG	28	0.24	-269.19

Supplementary Table 17: Aptamers generated from truncation with GA and RaptScore from dataset C. A 30-nt aptamer was truncated to generate aptamers with lengths ranging from 20 and 22 nucleotides. RaptScore and Relative activities are shown. Relative activity is defined as the binding activity scaled relative to the full-length aptamer. The sequences correspond to those presented in Figure 4C.

ID	Sequences	Length	Relative Activity	RaptScore
len_20	GGCCAAGGTTGCCGGCTTCA	20	83.10	-93.60
len_22	GGCCAAGGTTGCCGGCCATGCA	22	72.44	-115.09

Supplementary Table 18: Evaluation results of sequences generated by RaptGen for Dataset D. RaptScore and Relative activities are shown. Relative activity data is taken from reported values in the RaptGen study. The sequences correspond to those presented in Figure 5A.

ID	Sequences	Length	Relative Activity	Normalized RaptScore
A_L20_GMM_0	CTCGAGATTCTGAGGGTTCTGCATA	25	-0.7	-1.84
A_L20_GMM_1	AAGAAATTAGATACTAGTAAATACGACATA	30	-4.9	-3.82
A_L20_GMM_2	TGTGCGGATATGCTGAGTTTCTGC	24	-4.9	-4.20
A_L20_GMM_3	AACGAGAGATACAACCTGTGCGTGCC	26	8.8	-3.78
A_L20_GMM_4	ATCGAGAGATGGTAGACCCTGTGCG	25	-2.7	-1.61
A_L20_GMM_5	CAAAGACGTGCAAGCCTCGTCTACGGT	27	-1.5	-4.33
A_L20_GMM_6	TCTGAGGGTCCTGACAACAGAAACA	25	-0.7	-4.33
A_L20_GMM_7	TACGAGAGATGTAGCCTGTATGTGCT	26	11.9	-3.64
A_L20_GMM_8	CAGTAAGGTTTCGACACTCTTCTACTTA	28	-2.4	-4.19
A_L20_GMM_9	AACGAGAGATGTTGCCTGCGTGTTGC	26	4.0	-3.47
A_L25_GMM_0	CATCAATAATAAGAATAACAAAATTTCAAA	30	-1.1	-3.54
A_L25_GMM_1	CAACTACGAGAGATGTAGCCTGGA	24	-6.1	-4.25
A_L25_GMM_2	GTAGAGATTCTGAGGGTTCTCCCGCTCC	28	103.6	-1.87
A_L25_GMM_3	GGCGGTGATGTAGAAACGGTTGAGGTTAA	29	-2.1	-3.96
A_L25_GMM_4	ATACGAGAGATGTAGCCTGTGTCGTAGAA	29	-4.0	-2.76
A_L25_GMM_5	AACGAGAGATGGTAGACCGTTGTGGAT	27	63.2	-1.88
A_L25_GMM_6	CACGCGGGTTTCACACTCTATGAATGA	27	-0.1	-4.14
A_L25_GMM_7	CGTAGGGAATCTGAGGGGTCTCCCGCCCT	29	21.5	-3.58
A_L25_GMM_8	AACGGTAACACGTGCAAGCCTGTTATTAT	29	-4.3	-3.64
A_L25_GMM_9	ATTACGAGAGATACAGCCTATATCGTAGCA	30	15.4	-3.35
A_GMM_0	AACGAGAGATGGTAGACCTATCTTTTAGCC	30	79.0	-2.11
A_GMM_1	GTAGAGATTCTGAGGGTTCTCCTGCTATA	29	107.1	-1.83
A_GMM_2	TTTTATAAAAAAGTGTTTAAAAAAGATTCA	30	-3.6	-3.67
A_GMM_3	GTAGAAATTACGAGAGATGTCGCCTTTGA	29	7.0	-3.79
A_GMM_4	GGGGGTGCAGTAGAATTGTCGAGTTTCTG	29	15.9	-4.01
A_GMM_5	AATACCCGGGGTTTTCACACATATAATTCA	30	-0.8	-3.96
A_GMM_6	ATACGAGAGATGTAGCCTTTTTTCTGACTT	30	42.2	-2.89
A_GMM_7	AGTACGAGAGATACAGCCTTTTTCCTGCTT	30	30.9	-3.32
A_GMM_8	GGTAGCAGATGCTGAGGGGTCTCCTGATG C	30	33.8	-3.86
A_GMM_9	GTCGAGATTCTGAGGGTTCTCCTGTTAACC	30	74.3	-2.01
A_BO_0	GTAGAGATTCTGAGGGTTCTCCTGTTGACC	30	102.5	-1.95
A_BO_1	GTTGAGATTCTGAGGGTTCTCCTGTTGCCC	30	101.2	-1.96
A_BO_2	AACAAGAGATGGTAGACCTATCTCTTACCC	30	69.4	-2.41
A_BO_3	GTAGAGATTCTGAGGGTTCTCCTGTTGCTA	30	100.6	-1.94
A_BO_4	AACGAGAGATGGTAGACCTATCTTTTAGCC	30	76.0	-2.11
A_BO_5	GTCGAGATTCTGAGGGTTCTCCTGGTGACC	30	74.6	-2.00
A_BO_6	AACGAGAGATGGTAGACCTATTTTTTAGTC	30	73.1	-2.11
A_BO_7	AATGAGATTCTGAGGGGTCTCCTGTTGCCA	30	95.1	-1.87
A_BO_8	ATACGAGAGATGTAGCCTTTTTTCTTACTT	30	40.8	-2.79
A_BO_9	ATACGAGAGATGTAGCCTTTTTACCGACCT	30	15.0	-2.74

Supplementary Table 19: Evaluation results of sequences generated by RaptGen for Dataset E. RaptScore and Relative activities are shown. Relative activity data is taken from reported values in the RaptGen study. The sequences correspond to those presented in Figure 5B.

ID	Sequences	Length	Relative Activity	Normalized RaptScore
B_L30_GMM_0	ACUCACAUUACGUAAAAAUCGCCCCUACC	29	6.4	-4.11
B_L30_GMM_1	UGGAUACGCAAAAGCUCCCCCUGCCUUACA	30	-16.1	-4.26
B_L30_GMM_2	UAAAACAGCUGCCCCCCCCCCAUCUGACCCGACGACCAAC	40	-14.6	-4.37
B_L30_GMM_3	UACGACCAGCUACGCAACAGUUCUCCCUGCCUGA	34	-22.9	-4.18
B_L30_GMM_4	UUCGCUACGCGAAAGUUCCCCCCGCCUGGCG	31	-12.4	-4.18
B_L30_GMM_5	UUCGCUACGCUAAAGCUCUCCCAGCCUGGCG	31	-21.6	-4.23
B_L30_GMM_6	ACUCACAUUACGCAAAACUCGCCCCUGCC	29	86.9	-3.87
B_L30_GMM_7	UCUACGUCAAACUCGCCCCGACCUGGCG	28	-21.9	-4.05
B_L30_GMM_8	ACACACAUUACGCGAAACUCGCCCCCGCC	29	183.3	-4.01
B_L30_GMM_9	UUCGCUACGCAAAAGUUCCCCCUGCCUGGCG	31	-19.7	-4.32
B_L35_GMM_0	UGCUCGACACUACGCAACACUUCCCCCUGCCUGACAUA	38	-12.1	-4.11
B_L35_GMM_1	UCCGCACGCCAGCGCACAUUACGUAAAGAUCGCCCUACC	39	-5.2	-4.02
B_L35_GMM_2	UGCACGACGCUACGCCAAACUCCCCCCGGCCUGAUAAA	38	57.7	-3.92
B_L35_GMM_3	UAACACAGCCCCACCCCUCGGACGACCGGAGGAAUAAAAA	40	-14.9	-3.86
B_L35_GMM_4	UGCUCUAGCUACGCGAAAAUCCCCCCCGCCUGCAUGCGCACA	42	23.0	-4.17
B_L35_GMM_5	UCCGCCCGCCAGCGCACAUUACGCAAAGAUCGCCCUGCC	39	-25.7	-3.86
B_L35_GMM_6	UUCGCUAGCGUACGCAAAACUCCCCCCUGCCUUGCAUGCGCUUA	44	-16.9	-4.35
B_L35_GMM_7	UACCACGCCCGCGCACAUUACGCGAAGAUCGCCCCGCC	38	-22.4	-3.88
B_L35_GMM_8	UCCGCCAACCCCCCCCCUCCCCCACCCCCCCACCUCCAAA	40	-22.5	-4.44
B_L35_GMM_9	UGCGCGUCACUACGCUAAAAUCCCCCCAGCCUGACAAA	38	-10.0	-4.18
B_GMM_0	UACACACCCCCCACCCCCCCCCGCCCCCCCCCCCCCAAA	40	-13.2	-4.60
B_GMM_1	UCUCUGCUUACGCCAAAAUUCCCCCGGCCUAGCUUGGCUCGCUC	44	21.9	-4.33
B_GMM_2	ACGCCUACGCCAAAAGCCCCCCAGCCUGGCUUGGCGCGCAC	41	-9.3	-4.19
B_GMM_3	UGCGCGACCGCGCGCACAUUACGCGAAACUCCCCCCCGCC	40	74.2	-3.46
B_GMM_4	UGCGCGCCCAGCGCACAUUACGUAAAACUCCCCCCUACC	39	229.1	-4.02
B_GMM_5	UCGCCCUGCGCACAUAACUACGCAAAACUCCCCCCCGCCA	40	-30.5	-4.04
B_GMM_6	UCGCCUACGCAAAAAACUCCCCCUGCCUGUAUCACUCAC	39	-28.5	-4.21
B_GMM_7	UGCGCUACUCUACGCUAAACUCCCCCCAGCCUGGAAA	37	-7.3	-4.29
B_GMM_8	UGCGCGCCCGAGCGCACAUUACGCAAAAUCCCCCCUGCC	39	190.7	-3.72
B_GMM_9	UGCGAUACGCGAAACGCUCCCCCGCCUUCCUAG	33	-7.3	-4.10
B_BO_0	UGCGCGAGCCGCGCCAUUACGCAACACUCGCCCCCUGCC	39	5.6	-4.06
B_BO_1	UGCGCGAGGCCGCGCCAUUACGUAACACUCGACCCCUACC	40	3.2	-4.23
B_BO_2	UGCGCGACCCGCGCCAUUACGCAACACCCCCCCUGCC	37	79.4	-4.02
B_BO_3	UGCGCGAGCCGCGCACAUUACGUAAAAAAUCGACCCCUACC	41	13.5	-3.95
B_BO_4	UGCGCGCCCAGCGCACAUUACGCAACACUCCCCCCUGCC	39	231.0	-3.82
B_BO_5	UGCGCGCCCGAGCGCACAUUACGUAAAACUCCCCCCUACC	40	245.2	-3.97
B_BO_6	UGCGCGACCGCGCGCACAUUACGCAACACCCCCCCUGCC	39	93.7	-3.55
B_BO_7	UGCGCGAGCAGCGCCAUUACGUAACACUCGCCCCCUACC	39	45.2	-4.21
B_BO_8	UGCGCGCCCGCGCGCACAUUACGCAACACUCCCCCCUGCC	40	134.9	-3.65
B_BO_9	UGCGCGCCCAGCGCACAUUACGUAAAAACCCCCCUACC	38	151.6	-3.77
